# Reactive oxygen species in iridium-based OER catalysts†
†Electronic supplementary information (ESI) available. See DOI: 10.1039/c6sc01860b



**DOI:** 10.1039/c6sc01860b

**Published:** 2016-07-19

**Authors:** Verena Pfeifer, Travis E. Jones, Sabine Wrabetz, Cyriac Massué, Juan J. Velasco Vélez, Rosa Arrigo, Michael Scherzer, Simone Piccinin, Michael Hävecker, Axel Knop-Gericke, Robert Schlögl

**Affiliations:** a Department of Inorganic Chemistry , Fritz-Haber-Institut der Max-Planck-Gesellschaft , Faradayweg 4-6 , Berlin , 14195 , Germany . Email: trjones@fhi-berlin.mpg.de; b Catalysis for Energy , Group EM-GKAT , Helmholtz-Zentrum Berlin für Materialien und Energie GmbH , Elektronenspeicherring BESSY II , Albert-Einstein-Str. 15 , Berlin , 12489 , Germany; c Department of Heterogeneous Reactions , Max-Planck-Institut für Chemische Energiekonversion , Stiftstr. 34-36 , Mülheim a. d. Ruhr , 45470 , Germany; d Diamond Light Source Ltd. , Harwell Science & Innovation Campus , Didcot , Oxfordshire OX 11 0DE , UK; e Consiglio Nazionale delle Ricerche – Istituto Officina dei Materiali , c/o SISSA, Via Bonomea 265 , Trieste , 34136 , Italy

## Abstract

Tremendous effort has been devoted towards elucidating the fundamental reasons for the higher activity of hydrated amorphous Ir^III/IV^ oxyhydroxides (IrO_*x*_) in the oxygen evolution reaction (OER) in comparison with their crystalline counterpart, rutile-type IrO_2_, by focusing on the metal oxidation state. Here we demonstrate that, through an analogy to photosystem II, the nature of this reactive species is not solely a property of the metal but is intimately tied to the electronic structure of oxygen. We use a combination of synchrotron-based X-ray photoemission and absorption spectroscopies, *ab initio* calculations, and microcalorimetry to show that holes in the O 2p states in amorphous IrO_*x*_ give rise to a weakly bound oxygen that is extremely susceptible to nucleophilic attack, reacting stoichiometrically with CO already at room temperature. As such, we expect this species to play the critical role of the electrophilic oxygen involved in O–O bond formation in the electrocatalytic OER on IrO_*x*_. We propose that the dynamic nature of the Ir framework in amorphous IrO_*x*_ imparts the flexibility in Ir oxidation state required for the formation of this active electrophilic oxygen.

## Abstract 2

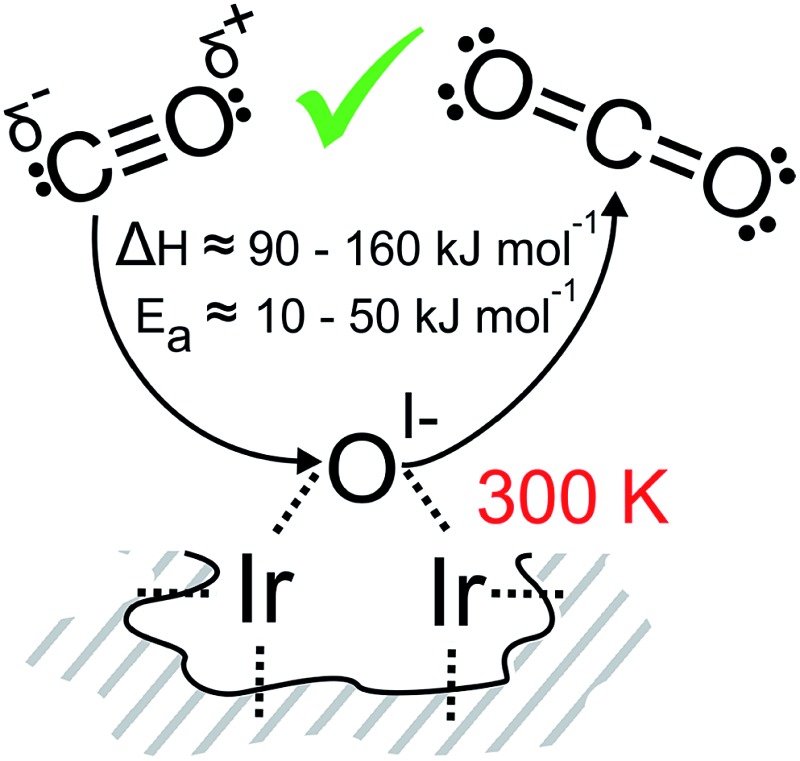
Exceptional reactivity of electrophilic oxygen species in highly OER-active Ir^III/IV^ oxyhydroxides is evidenced by room temperature CO oxidation.

Ir-based materials are promising candidates to catalyze the oxygen evolution reaction (OER) in acidic media. Iridium oxides, both anhydrous rutile-type IrO_2_ and hydrated X-ray amorphous forms, have been widely considered as possible OER catalysts.^1–8^ Of these, the amorphous and nanostructured Ir-based catalysts have been shown to exhibit higher OER activities than rutile-type IrO_2_.^9,10^ Although numerous studies exist on such amorphous structures, and activity descriptors like mixed iridium oxidation states^11,12^ and the surface OH-concentration^13^ have been proposed, a convincing explanation for the enhanced activity of amorphous structures over crystalline ones is lacking. While many studies concentrated on investigating active metal centers and the iridium oxidation state,^11,12,14^ by analogy with the well-studied biological water splitting, an investigation of preactivated oxygen species contained in OER catalysts seems appropriate.

Oxygen preactivated for O–O bond formation is essential in photocatalytic water splitting over the tetranuclear Mn water oxidation complex (WOC) of photosystem II (PS II).^15,16^ A proposed mechanism of O–O bond formation in PS II is the nucleophilic attack of oxygen adsorbed in the WOC (O*) by a (preadsorbed) water molecule/hydroxide:^17,18^

1 WOC–O* + H_2_O_(ads)_ → WOC–O–O–H + H^+^ + e^–^.

In this mechanism water acts as the nucleophile, donating electrons to the reactive oxygen, which can alternatively be formulated WOC≡O^+^ to emphasize its susceptibility to nucleophilic attack.^15^ Within this mechanism, the ability of Mn to accommodate different oxidation states enables the preparation of the electrophilic oxygen.

Nucleophilic attack of adsorbed oxygen by water/hydroxide has also been proposed to describe the mechanism of O–O bond formation on transition metal oxides during the OER.^4,19^ For iridium oxide, the importance of reactive oxygen is evident from isotope labeling experiments,^5^ which suggest the outer layers of the catalyst are involved directly in the electrocatalytic OER. Thus, O–O bond formation can be written as:

2 IrO_*x*_O_(ads)_ + H_2_O → IrO_*x*_–O–O–H + H^+^ + e^–^,

with IrO_*x*_O_(ads)_ representing the iridium oxide matrix with an adsorbed oxygen. The oxidation state of the surrounding Ir metal centers must be flexible to allow this adsorbed oxygen to become an electrophilic species that is susceptible to nucleophilic attack. In line with this assumption, our previous study revealed that highly electrochemically active X-ray amorphous iridium oxides contain electronic defects in their cationic (Ir^III^) and anionic frameworks (O^I–^) that are nearly absent in less active rutile-type IrO_2_.^10^ The holes in the O 2p states of IrO_*x*_ may make the O^I–^ an electrophile that, in analogy to the biological cycle, may lead to the enhanced OER activity of amorphous IrO_*x*_. To test this hypothesis, we need to understand the chemistry of these O^I–^ species.

A powerful means of uncovering the chemistry of the O^I–^ is through CO oxidation, a reaction that has proven essential in developing a fundamental understanding of heterogeneous processes.^20^ Herein we use this probe to test if the O^I–^ in X-ray amorphous IrO_*x*_ can act as a strong electrophile. If this species is sufficiently electrophilic, we expect it to readily react with CO by accommodating the electron density of the partially negatively charged carbon atom of CO.^20,21^

The sample investigated is a commercially available, hydrated, X-ray amorphous Ir^III/IV^ oxyhydroxide (IrO_*x*_, Premion, AlfaAesar). In a control experiment, a less active, crystalline rutile-type IrO_2_ (Sigma Aldrich) was examined. The properties of both materials are described in ref. 10. Therein, we showed that O^I–^ is identifiable by an excitation energy resonance at 529 eV in the near-edge X-ray absorption fine structure (NEXAFS) of the O K-edge that is not present in pristine rutile-type IrO_2_. Herein, the spectroscopic features corresponding to these O^I–^ species were monitored by quasi *in situ* X-ray photoemission spectroscopy (XPS) and NEXAFS before and after CO exposure to observe whether O^I–^ reacts with CO during titration of the IrO_*x*_ surface. This approach ensures high quality artifact free oxygen spectra by preventing alterations of the sample during air exposure between measurements and CO exposure and eliminating gas phase contributions of CO and CO_2_ to the oxygen absorption spectra. The results are compared to theoretical calculations and a microcalorimetric analysis.

In a preliminary experiment, we tested the general activity of the two iridium oxide powders towards a stoichiometric oxidation of CO to CO_2_ at room temperature and atmospheric pressure in a standard flow-through reactor (details in ESI†). To ensure reaction of CO was exclusively with active oxygen from the iridium oxide samples, we did not add O_2_ to the gas feed and had oxygen filters attached to the gas dosing lines.

We observe that introducing CO into the reactor filled with rutile-type IrO_2_ leads to no change in the CO_2_ signal (Fig. 1). In contrast, when introducing CO into the reactor loaded with IrO_*x*_, the CO_2_ signal shows a sharp increase followed by a decline towards the baseline (Fig. 1). Such full CO conversion at ≈300 K is extremely unusual, having only been observed in a few novel systems, such as oxide-supported gold catalysts,^22^ and reveals the high reactivity of the IrO_*x*_. As there is no external oxygen source available in the present experiment, the effluent CO_2_ must be due to the stoichiometric reaction of CO with an oxygen species present in IrO_*x*_ and nearly absent from the rutile-type powder. Because only IrO_*x*_ has an abundance of O^I–^, this type of oxygen is a likely candidate for that active in CO oxidation. To test this hypothesis, we employ XPS/NEXAFS and monitor the regions characteristic for O^I–^ (≈529 eV).

**Fig. 1:**
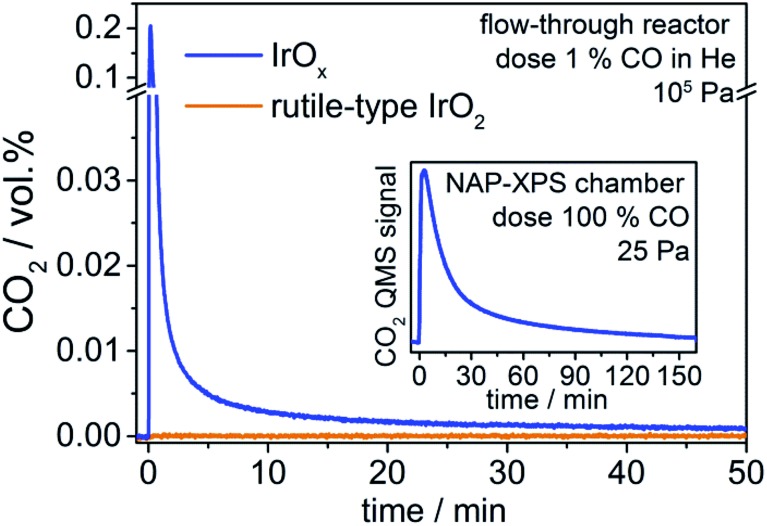
CO_2_ concentration in effluent gas stream over time after CO is introduced (0 min) in He stream (100 mL min^–1^ CO : He 1 : 99, 298 K) for IrO_*x*_ and rutile-type IrO_2_. IrO_*x*_ in contact with CO results in up to 0.2 vol% CO_2_ in the effluent gas stream. The inset shows the QMS signal for CO_2_ during the CO exposure of the IrO_*x*_ sample in the near-ambient-pressure XPS chamber.

In a quasi *in situ* experiment, we first measured the powder in vacuum as received (base pressure 10^–6^ Pa). We then exposed the IrO_*x*_ to pure CO in the NAP-XPS chamber (25 Pa, 2 mL min^–1^) for 3 h and observed a CO_2_ profile similar to that seen in the standard reactor (see inset Fig. 1). Finally, we evacuated the chamber and without air contact measured the sample after CO (details see ESI†).

When comparing the XPS core lines recorded before and after CO exposure, the Ir 4f spectra show no obvious changes (Fig. S3†). In contrast, the intensity at low binding energies (529–530 eV) in the O 1s spectrum is slightly reduced after CO exposure (Fig. S3†), indicating the potential loss of O^I–^. The corresponding O K-edge NEXAFS collected in the surface sensitive Auger electron yield mode (AEY) corroborate this finding. Here we see CO exposure leads to a loss in intensity at excitation energies around 529 eV (Fig. 2). A comparison of the difference spectrum of the O K-edges before and after CO exposure with that calculated for O^I–^ (see Fig. 2) supports this conclusion, suggesting O^I–^ is consumed from IrO_*x*_ by oxidizing CO to CO_2_ at room temperature.

**Fig. 2:**
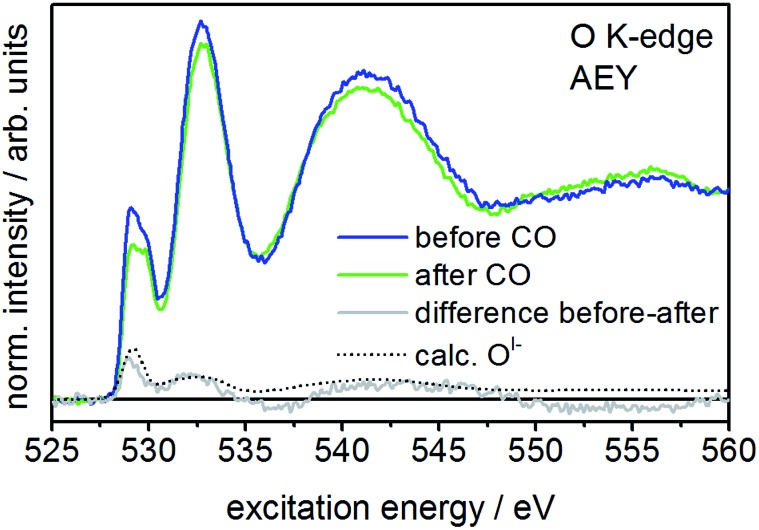
O K-edge of IrO_*x*_ measured in Auger electron yield (AEY) mode before and after CO exposure (25 Pa, 2 mL min^–1^ CO, 298 K). The difference spectrum shows the considerable decrease of the 529 eV feature and is in good agreement with the calculated spectrum of O^I–^ species.

Interestingly, more bulk sensitive total electron yield (TEY) measurements of the O K-edge also show reduced 529 eV intensity after CO exposure (see Fig. S5†). Hence, subsurface O^I–^ species migrating to the surface also appear to be involved in the reaction with CO. This observation alludes to the dynamic mobility of O^I–^ in the porous structure^10^ of IrO_*x*_. Nevertheless, since the CO_2_ signal in the standard reactor and the NAP-XPS chamber declines immediately after the initial increase, the reservoir of mobile O^I–^ species seems to be rapidly exhausted. However, since the 529 eV component does not vanish completely in either AEY and TEY, part of the O^I–^ seem unavailable for reaction with CO over the time scale of our experiment.

Though comparison of our theoretical and experimental O K-edge spectra supports the conclusion that O^I–^ participates in stoichiometric room temperature CO oxidation, we have not demonstrated such a reaction is feasible. To do so, we begin by considering the thermodynamics of the reaction of gas phase CO with oxygen, in oxidation state n (O^*n*–^), in an IrO_*x*_ matrix (IrO_*x*_O^*n*–^):

3 IrO_*x*_O^*n*–^ + CO_(g)_ → IrO_*x*_ + CO_2(g)_ + *n*e^–^.

The liberated *n*e^–^ may reduce adjacent Ir or O. We have assumed the product desorbs from the surface after forming, an assumption we will show to be valid. It is then straightforward to compute the heat of reaction for this process for different types of oxygen species by way of DFT (see ESI† for details), as the energies of the gas phase reactant and product are constants and only the adsorption energy of the oxygen species involved in the reaction changes the heat evolved (using the positive sign convention of microcalorimetry). And while we do not know the structure of the amorphous IrO_*x*_, we can use model systems to gain useful insights into the relationships between the electronic structure of oxygen and its adsorption energy.

To investigate the reaction of CO_(g)_ with surfaces species, we employed IrO_2_ (110) and IrO_2_ (113) surfaces as model structures, using both oxygen-terminated and partially reduced (110) surfaces along with a partially reduced (113) surface (see ESI† for full details). Both types of surface have under coordinated O atoms whose simulated O K-edge spectra agree with that measured for O^I–^ (see ESI†). The reduced surfaces also have under coordinated Ir atoms that can adsorb CO with adsorption energies of ≈180–210 kJ mol^–1^ (see ESI†).

As a first consideration, we computed the minimum energy path for the reaction of CO_(g)_ with an oxygen-terminated (110) surface. This surface contains three unique types of oxygen, an O^II–^ like species, an O^I–^ like species, and oxygen on the coordinatively unsaturated site (CUS), the last of which was not observed experimentally (Fig. S14†). The O^I–^ has a barrier for reaction (3), ≈10 kJ mol^–1^, that is slightly larger than *k*_B_ *T* at room temperature, and the reaction is ≈90 kJ mol^–1^ exothermic. In contrast, the activation energy associated with the reaction of CO_(g)_ directly with O^II–^ is significantly larger than the energy available at room temperature, 170 kJ mol^–1^, and is exothermic by ≈50 kJ mol^–1^.

To test the reaction of adsorbed CO with an O^I–^ like species, we computed minimum energy paths on the partially reduced (113) surface (see ESI† for details). In this case, CO oxidation by an O^I–^ has a slightly higher barrier, 50 kJ mol^–1^, than was observed on the fully oxidized (110) surface. Also, the initial product on the reduced surface is a strongly adsorbed CO_2_, not CO_2(g)_. Overall reaction (3) is ≈160 kJ mol^–1^ exothermic on the reduced (113) surface, which is in agreement with the 165 kJ mol^–1^ computed for the reaction of CO_(g)_ with O^I–^ on a partially reduced (110) surface with an Ir vacancy.

These results suggest that, if present, surface O^I–^ species are highly electrophilic, with the fully oxidized surface perhaps showing higher reactivity. These electrophilic anionic defects are, in fact, predicted to be so reactive that they should not persist except under ultra-high vacuum, which may explain the their absence in rutile-type IrO_2_ loaded from air^10^ and the lack of CO_2_ formation when rutile-type IrO_2_ is exposed to CO (see Fig. 1). However, we know from the TEY measurements that, unlike the rutile-type oxide, the amorphous oxides contain a significant concentration of potentially mobile O^I–^ in the subsurface/bulk region. These subsurface species are depleted during CO oxidation (see Fig. S5†). If we assume that once on the surface any O^I–^ will behave in a manner analogous to the aforementioned surface species, we can focus on the bulk as a thermodynamic source of oxygen and compute its associated heat of reaction with CO_(g)_.

We find that reaction with the bulk O^II–^ of rutile-type IrO_2_ is thermoneutral, whereas reaction with bulk O^I–^ species^10^ is 110–120 kJ mol^–1^ exothermic, depending on the atomic structure around the anionic defect. As with the surfaces, we see that bulk O^I–^ species are more weakly bound, hence more electrophilic, than the bulk O^II–^. These results are summarized schematically in Fig. 3.

**Fig. 3:**
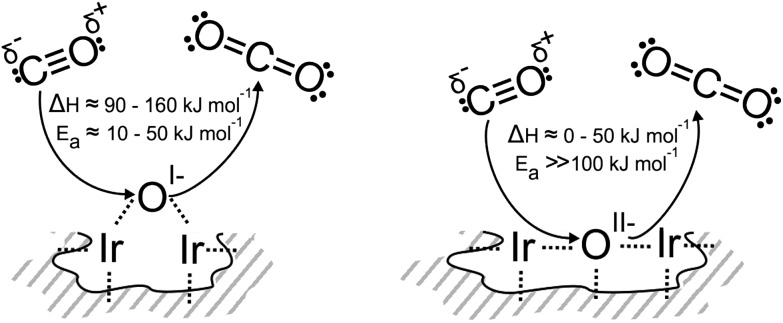
The schemes summarize the CO titration of different oxygen species in iridium oxides with respect to their calculated activation barrier and reaction enthalpies. Exothermic processes are given by positive values (positive sign criterion of microcalorimetry). Energetically, the reaction of CO with O^I–^ seems feasible at room temperature while reaction with O^II–^ does not.

When the heats of reaction for all the oxygen species studied in this work are considered, we find that the O^I–^ type generally gives rise to higher heats of reaction than the formally O^II–^. Remarkably, in the absence of major structural relaxations of the surface after reaction, there is a rough linear relationship between the position of the white line in the calculated O K-edge spectrum of an oxygen species and its corresponding heat of reaction with CO (see Fig. S15†). Thus, the O K-edge spectra appear to offer a means of probing the electrophilic character of the oxygen. From this measure, we would predict the heat of reaction of the dominant O^I–^ species present in our experiments (O K ≈ 529 eV) is 100 kJ mol^–1^.

To validate the calculated heats of reaction for room temperature CO oxidation on IrO_*x*_, we titrated IrO_*x*_ with CO at 313 K using *in situ* microcalorimetry.^23^ A gas phase analysis after the experiment confirmed the predominant presence of CO_2(g)_, supporting our assumption in eqn (3) and alluding to complete oxidation of the dosed CO (see Fig. S11†). Hence, in the determination of the differential heat of reaction, we took into account the entire dosed CO. Doing so, and averaging over three measurements, we obtain an exothermic heat of reaction of 125 kJ mol^–1^ ± 40 kJ mol^–1^ for the 2 mmol g^–1^ of reacted CO (see Fig. 4). These measured differential heats of reaction agree with those calculated for CO_(g)_ oxidation by O^I–^ species (see Fig. 4), whereas they are much larger than the thermoneutral reaction of CO_(g)_ with O^II–^. In fact, the mean measured value (125 kJ mol^–1^) is similar to the heats calculated for reaction with bulk O^I–^, while the upper and lower ends of the standard deviation window agree with the calculated reaction enthalpies for CO_(g)_ with O^I–^ on the partially reduced (113) and fully oxidized (110) surfaces, respectively. In addition, we observe a broadening of the thermosignal time profiles with increasing amount of reacted CO (see Fig. S8–S10†), which could be an indication for the involvement of subsurface O^I–^ liberated by the reaction heat evolved in the surface region.

**Fig. 4:**
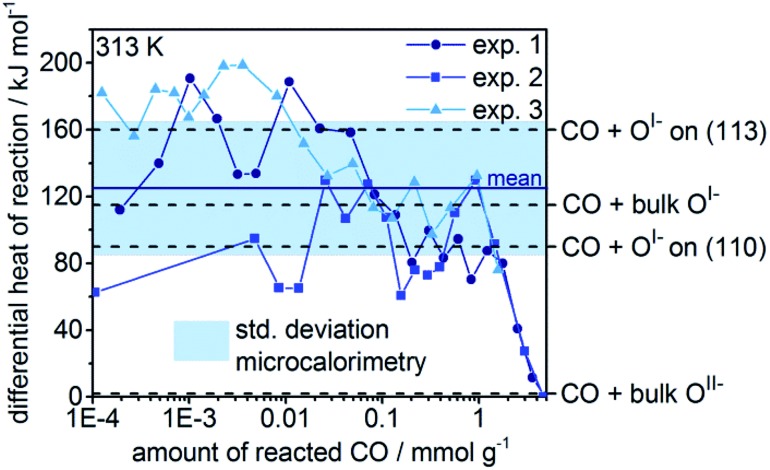
Differential heats over the amount of reacted CO in the microcalorimeter for three experiments with IrO_*x*_. An average heat of reaction of 125 kJ mol^–1^ ± 40 kJ mol^–1^ is determined. The dotted lines show the calculated heats of reaction for CO with different oxygen species of IrO_*x*_.

Hence, the calculations corroborate our assumption that during the exposure of IrO_*x*_ to CO, O^I–^ reacts with CO at room temperature. Combining our NEXAFS and microcalorimetric measurements with our theoretical calculations therefore suggests that the O^I–^ is a stoichiometric oxidant of CO to CO_2_, and therefore strongly electrophilic.

Now that we have demonstrated the reactivity of the O^I–^ species, it seems appropriate to consider their regeneration since for the OER cycle to be catalytic, reactive oxygen must be replenished. To test whether O^I–^ can be replenished on IrO_*x*_ we performed additional quasi *in situ* XPS and NEXAFS experiments. Following the CO titration, we exposed the sample successively to different gas atmospheres (25 Pa O_2_, 25 Pa H_2_O, and 25 Pa O_2_ : O_3_ (99 : 1)) in the NAP-XPS chamber for 20 min each to discern whether any of these gases can replenish the reactive oxygen sites. After each gas treatment, we evacuated the chamber and measured XPS and NEXAFS in UHV without allowing the samples to contact air.

We observed distinct changes in the O K-edge only after the exposure of the sample to the ozone-containing gas mixture. A comparison of the O K-edges after CO and after subsequent ozone exposure indicates a replenishment of O^I–^ by a more intense 529 eV feature after ozone exposure, see Fig. S6.† However, we found that the formally O^I–^ species replenished by ozone were not stable, as indicated by a reduction in the 529 eV resonance after 30 min in vacuum. This observation suggests that parts of the matrix stabilizing these O^I–^ species are lost during the oxidation of CO and cannot be restructured under such low pressure at room temperature. Nevertheless, ozone, as a source of atomic oxygen, seems to be capable of at least temporarily refilling the O^I–^ vacancies formed during reaction with CO. Hence, we have demonstrated that the replenishment of these reactive oxygen species is feasible, which makes them relevant for the catalytic OER cycle.

The local structure of the O^I–^ species considered in our work can be characterized as a µ_2_-oxo bridge, which can be seen to arise naturally when defects are introduced into the cationic framework of the parent rutile-type structure, thereby transforming µ_3_-oxo into µ_2_-oxo bridges. This process gives rise to holes in the O 2p states, due to the removal of Ir^IV^ and 4e^–^, making the µ_2_-oxo bridges charge deficient.^10,24^ Such µ_2_-oxo bridged atomic structures have been identified as key motifs in amorphous IrO_*x*_ thin films^25^ and as active centers in liquid-phase iridium oxidation catalysts^26^ by extended X-ray absorption fine structure (EXAFS) measurements, which are able to resolve the local atomic structure of amorphous materials. It is worth noting, however, that the electrophilic O^I–^ species seen in XPS and NEXAFS need not exclusively be due to such µ_2_-oxo bridges. Instead, they may also emerge when substituting an Ir^IV^ with a lower-valent element, such as Ni^II^,^13^ as the holes formed by replacing Ir^IV^ and 4e^–^ with Ni^II^ and 2e^–^ can localize on neighboring oxygen atoms. Thus, both types of environment, the under-coordinated and the lower-valent, can result in the emergence of an electron deficiency on oxygen. And while other potentially electron deficient oxygen species, in particular Ir=O, can also be imagined, we find no evidence for them in the O K-edge spectra of the rutile-type IrO_2_ or amorphous IrO_*x*_ samples, see Fig. S14.†

In conclusion, we showed that the O^I–^ species adsorbed on the surface and in the subsurface of OER-active Ir^III/IV^ oxyhydroxides are extremely electrophilic. By combining advanced experimental and theoretical techniques we found that this electrophilic oxygen can even oxidize CO to CO_2_ at room temperature, with a portion of the reacting oxygen coming from the subsurface region of the amorphous catalyst. The exceptional reactivity of the formally O^I–^ species is suspected to play a critical role in the OER by reacting with OH/H_2_O to form the OOH intermediate, which, when considering that weakly OER-active crystalline rutile-type IrO_2_ is nearly devoid of this reactive oxygen, explains the exceptional reactivity of amorphous Ir^III/IV^ oxyhydroxides. Like in biological water splitting, the electrophilic oxygen in Ir^III/IV^ oxyhydroxides is an optimal precursor site for the nucleophilic attack of (preadsorbed) water during the O–O bond formation. The creation of electrophilic oxygen in PS II is enabled by the easy accommodation of different oxidation states of the coordinated Mn ions. Likewise flexibility in the oxidation state for iridium is needed for electrophilic oxygen formation in iridium, which is apparently found in the Ir^III/IV^ oxyhydroxides.
