# Supplementary material for: Reactive oxygen species in iridium-based OER catalysts
Source: Chem Sci. 2016 Jul 19;7(11):6791–5. doi: 10.1039/c6sc01860b (PMC5134683; doi:10.1039/c6sc01860b)
Supplement: Supplementary file 1 [file SC-007-C6SC01860B-s001.pdf]

Electronic Supplementary Material (ESI) for Chemical Science.
This journal is © The Royal Society of Chemistry 2016

Supplementary Information to

Reactive oxygen species in iridium-based OER catalysts

Verena Pfeifer,[ab] Travis E. Jones,[a*] Sabine Wrabetz,[a] Cyriac Massué,[ac] Juan J. Velasco Vélez,[ac] Rosa Arrigo,[d] Michael Scherzer,[ac] Simone Piccinin,[e] Michael Hävecker,[ac] Axel Knop-Gericke[a] and Robert Schlögl[ac]

a *Department of Inorganic Chemistry, Fritz-Haber-Institut der Max-Planck-Gesellschaft, Faradayweg 4-6, Berlin, 14195, Germany. E-mail: trjones@fhi-berlin.mpg.de*

b *Catalysis for Energy, Group EM-GKAT, Helmholtz-Zentrum Berlin für Materialien und Energie GmbH, Elektronenspeicherring BESSY II, Albert-Einstein-Str. 15, Berlin, 12489, Germany*

c *Department of Heterogeneous Reactions, Max-Planck-Institut für Chemische Energiekonversion, Stiftstr. 34-36, Mülheim a. d. Ruhr, 45470, Germany*

d *Diamond Light Source Ltd., Harwell Science & Innovation Campus, Didcot, Oxfordshire OX 11 0DE, UK*

e *Consiglio Nazionale delle Ricerche - Instituto Officina dei Materiali, c/o SISSA, Via Bonomea 267, Trieste, 34136, Italy*

Contents

1 CO exposition of iridium oxides in flow-through reactor **3**

2 CO exposition IrO_x in Near-Ambient-Pressure XPS setup **4**

3 CO titration in SETARAM MS70 Calvet microcalorimeter **8**

4 Calculation details **11**

4.1 O K-edge spectra . . . 12

4.2 Heats of reaction . . . 14

4.3 Reactions on (110) . . . 16

4.4 Reactions on (113) . . . 17

1 CO exposition of iridium oxides in flow-through reactor

CO oxidation experiments over iridium oxide powders were first performed in a glass-lined steel, U-shaped tube reactor with an inner diameter of 5 mm. 25 mg of iridium oxide powder were diluted in 250 mg inert SiC powder with a particle diameter of 250 μm – 355 μm. Prior to CO exposure, the samples were dried in a He flow of 100 mL min^{-1} (controlled by Bronkhorst mass flow controllers) for 1 h at room temperature. The subsequent switch from inert He to a 1 % CO in He flow (100 mL min^{-1}) was realized via a 6-port switch valve (Valvo, Vici), which excluded dead volumes. The effluent gas was monitored on line with an X-Stream, Emerson/Rosemont gas analyzer to quantify CO and CO_2 concentrations. The catalyst temperature was recorded by an analog connection to the gas analyzer. To ensure gas purity, the CO line was equipped with a carbonyl remover (consisting of a tube filled with inert SiC heated to 573 K) as well as a CO_2-trap (crushed KOH filled cartridge). The He line had water as well as oxygen filters. The iridium oxide samples were an X-ray amorphous $Ir^{III/IV}$ oxyhydroxide (IrO_x, 99.99 % metals basis, AlfaAesar Premion®) and a crystalline rutile-type IrO_2 (99.9 % metals basis, Sigma Aldrich). Details on the materials' properties can be found elsewhere.[1]

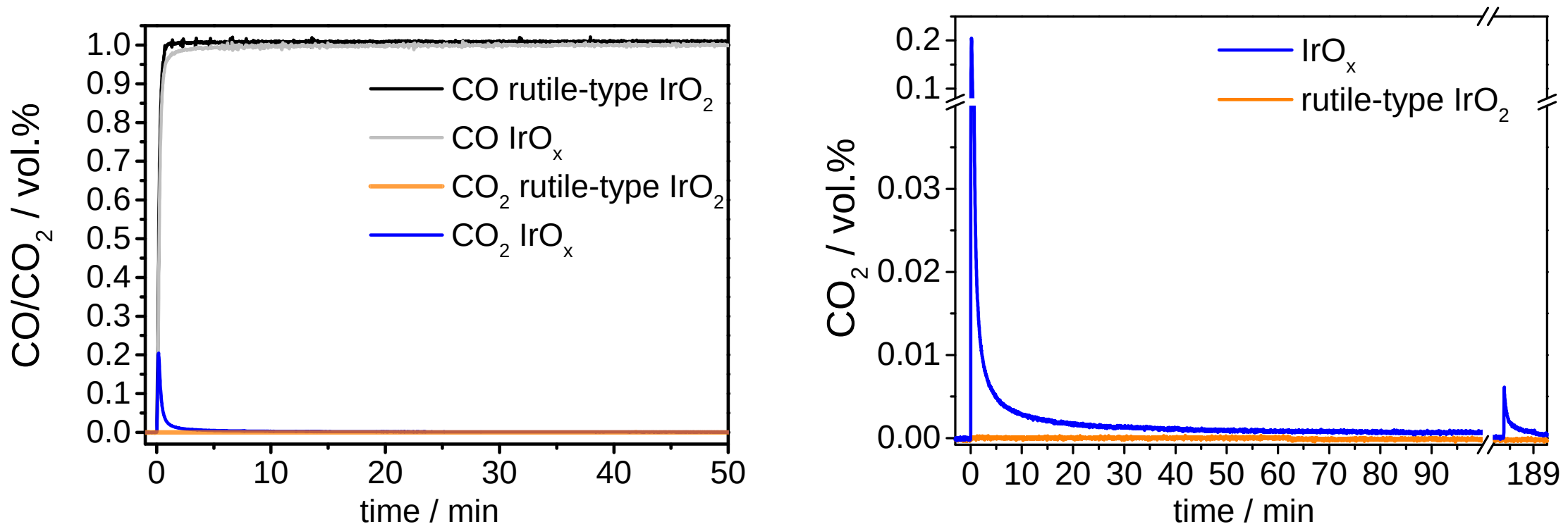

Figure S1: CO_2 concentration in effluent gas stream over time during CO dosage in inert gas stream (CO:He 1:99) for IrO_x and rutile-type IrO_2. The left graph shows both the CO and CO_2 traces for the two samples. The right graph shows only the CO_2 traces zoomed in and includes a second CO dosage after having exposed the sample to a He flow for 20 min after the first CO dosage. During the second CO dosage, starting at 183 min, the CO_2 concentration is two orders of magnitude smaller than during the first CO dosage, starting at 0 min.

Figure S1 shows that upon initial CO introduction into the reactor loaded with IrO_x, a strong CO_2 signal is measured that declines to a value close to the baseline within 50 min. In a second dosage of CO, after 20 min of pure He exposition, the recorded CO_2 signal of IrO_x is smaller by two orders of magnitude, which corroborates the assumption

that the majority of reactive oxygen surface species was consumed during the first CO exposure. For the rutile-type IrO_2 sample, no CO_2 is registered upon the exposition of the sample to CO (Figure S1).

2 CO exposition IrO_x in Near-Ambient-Pressure XPS setup

The near-ambient-pressure X-ray Photoemission Spectroscopy (NAP-XPS) system at the ISISS (Innovative Station for *In Situ* Spectroscopy) beamline[2] in the synchrotron facility BESSY II/HZB (Berlin, Germany) was used to perform quasi *in situ* photoemission and absorption experiments. The iridium oxide powder (IrO_x, 99.99 % metals basis, AlfaAesar Premion®) was pressed into a self-supporting pellet (40 mg, 3 t, 90 s, Ø = 8 mm). After a measurement of the sample as received in UHV ($\approx 10^{-6}$ Pa), the sample was exposed to 25 Pa CO at a flow rate of 2 mL min^{-1} for 3 h and afterwards measured again in UHV. The advantage of comparing spectra measured in vacuum is that there is no interference from the gas-phase contributions. This approach is especially important for the O K-edge, which would otherwise have a significant contribution from the gas-phase signals of CO and CO_2.
Successively, 25 Pa O_2 (3 mL min^{-1}), 25 Pa H_2O (3 mL min^{-1}) and 25 Pa O_2:O_3 (99:1) were dosed each for 20 min and NEXAFS was measured in between without air contact. For the ozone generation, a commercially available ozone generator TC-1KC was used. Oxygen was passed through Teflon tubing at a rate of 1 L min^{-1}. The effluent gas contained a mixture of ≈1 % ozone and 99 % molecular oxygen and was dosed into the measurement compartment via a leak valve.
In the XPS measurements, a pass energy (PE) of 10 eV and an exit slit setting of 111 μm was used, which led to an approximate resolution of 0.2 eV for the Ir 4f core line and 0.4 eV for the O 1s core line at 130 eV and 150 eV kinetic energy of the photoelectrons (KE), respectively. With the model of Tanuma *et al.*,[3] an inelastic mean free path (IMFP) of the photoelectrons of ≈0.4 nm is estimated. The binding energy calibration was carried out after evaluating each corresponding Fermi edge.
In NEXAFS, the photon energy was varied between 525 eV and 550 eV by a continuous movement of the monochromator. The Auger electron yield (AEY) of the O K-edge was collected with the electron spectrometer at a fixed KE of 385 eV (PE 50 eV). The probing depth of this measurement is ≈2 nm - 3 nm. Simultaneously, the total electron

yield (TEY) of the O K-edge was collected via a Faraday cup with an accelerating voltage applied. In this case the measurement depth is ≈5 nm - 10 nm.

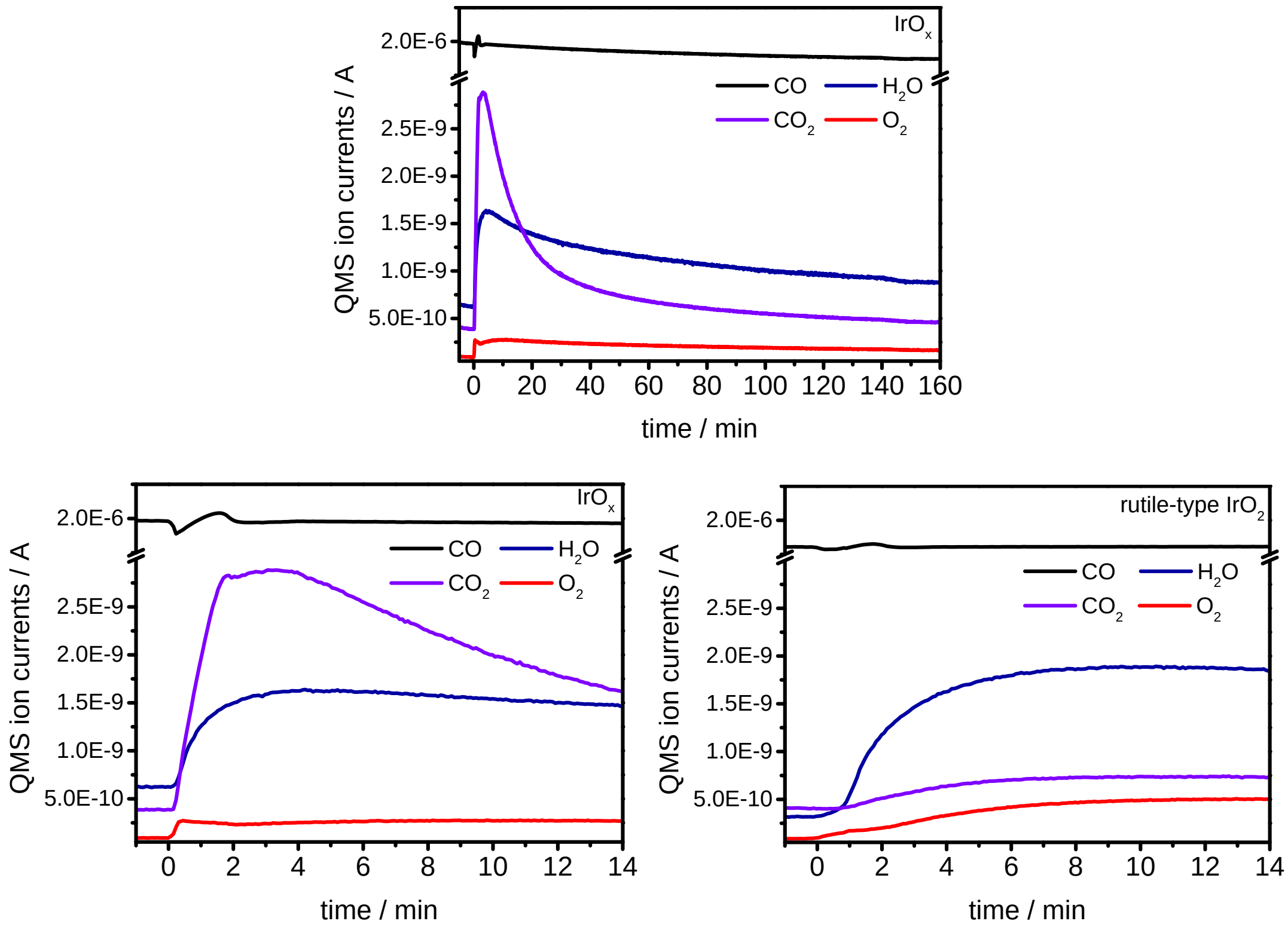

Figure S2: QMS traces of CO, CO_2, H_2O, and O_2 recorded in the NAP-XPS chamber during the CO exposure of the IrO_x (top, lower left (zoomed in)) and rutile-type IrO_2 (lower right). Over the course of the entire experiment of the IrO_x sample (top), the shape of the CO_2 profile for the IrO_x is considerably different to the traces of H_2O and O_2, confirming that the observed formation of CO_2 is not simply a reaction of CO with residual gas-phase oxygen or water. The figures in the lower part show a comparison of the active (IrO_x, left) and inactive (rutile-type IrO_2, right) samples. In both experiments, when introducing the sample at t = 0 min into the chamber filled with CO, also the H_2O and the O_2 traces increase since more surface area of the setup (i.e. load lock in which sample was stored) was exposed, which led to a slightly different background signal of the QMS traces. Nevertheless, it is evident that the change in the CO_2 trace of the IrO_x is not just a change in background signal, but the actual formation of CO_2.

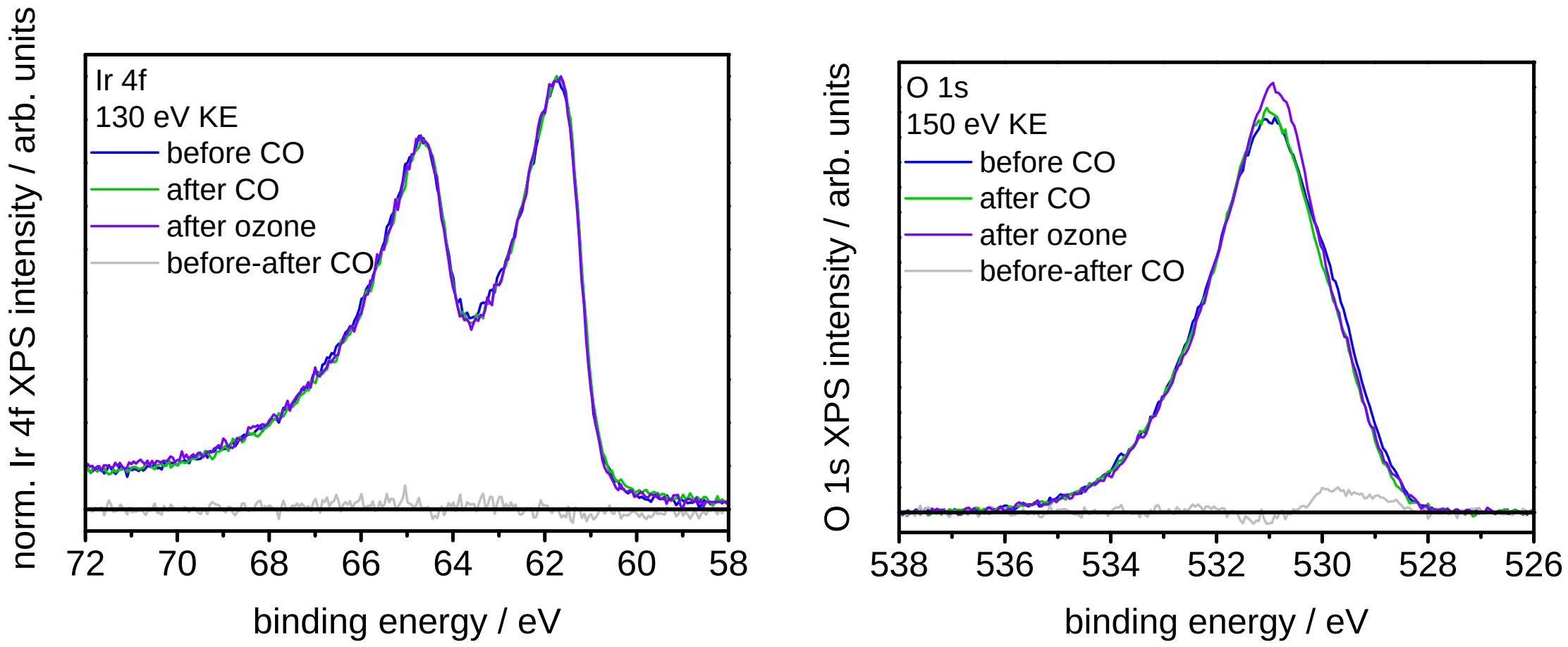

Figure S3: Ir 4f spectra (left) and O 1s spectra (right) of IrO_x before and after CO and after ozone exposure. While the Ir 4f spectrum is affected neither by CO nor by ozone, the intensity of the O 1s spectrum at low binding energies slightly decreases after CO exposure. The intensity increase in the O 1s spectrum at ≈531 eV after ozone exposure is most likely due to carbonate formation at the surface (compare to C 1s spectrum in Figure S4 (left)). This carbonate formation at the surface might mask the replenishment of O^{I-} in the top layer of IrO_x.

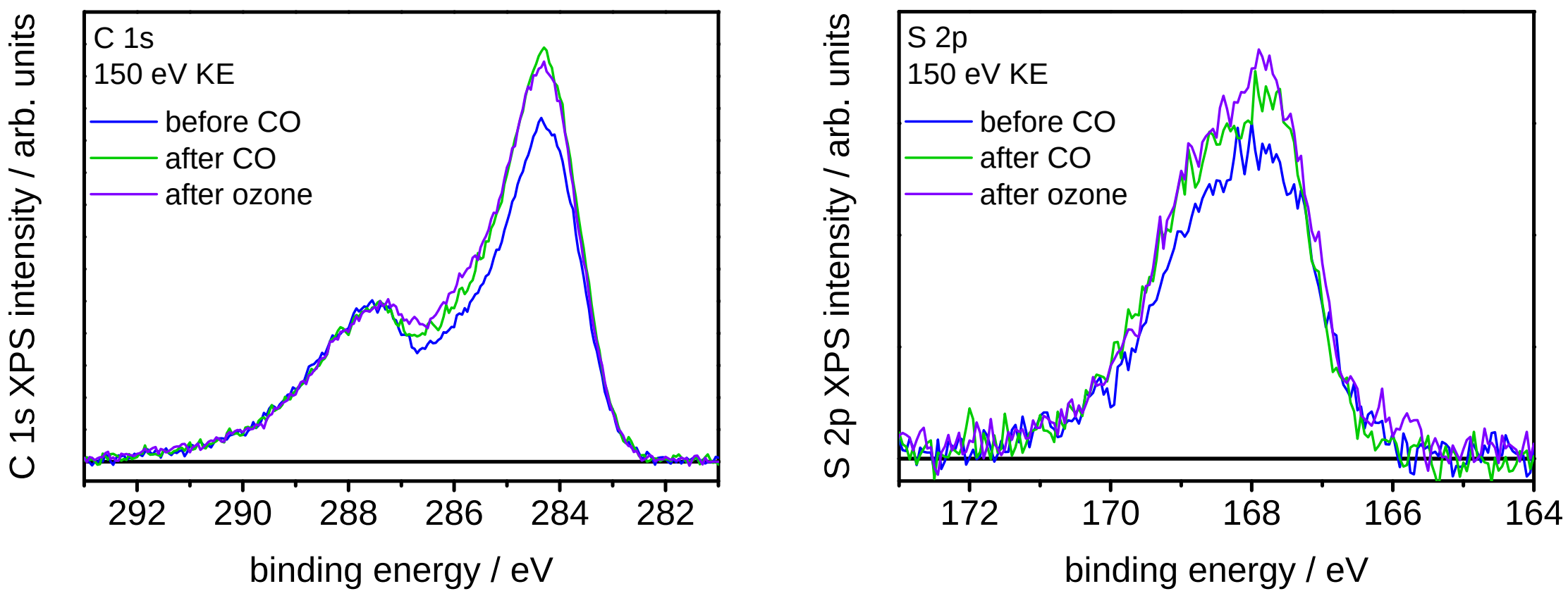

Figure S4: C 1s spectra (left) and S 2p spectra (right) of IrO_x before and after CO and after ozone exposure. After the CO exposure, mainly the intensity at around 284.5 eV corresponding to graphitic carbon increases, a phenomenon commonly observed in near-ambient-pressure XPS chambers due to the "high" base pressure (10^{-6} Pa) of such systems when compared to UHV-only systems (10^{-8} Pa).[4] This phenomenon also occurs when keeping the sample in vacuum without dosing CO as shown in Figure S7. After the ozone exposure a slight increase of intensity at around 286 eV may be seen due to oxidation of carbon forming carbonates (as also indicated in the O 1s spectrum Figure S3) during the presence of ozone. The S 2p spectra show that the amount of S on the sample is slightly increasing during the course of the experiment. Since similar concentrations of C and S are observed before and after ozone, it can be excluded that the decrease/increase of the 529 eV feature after CO/after ozone is simply the result of carbon or sulfur species covering the O^{I-} species.

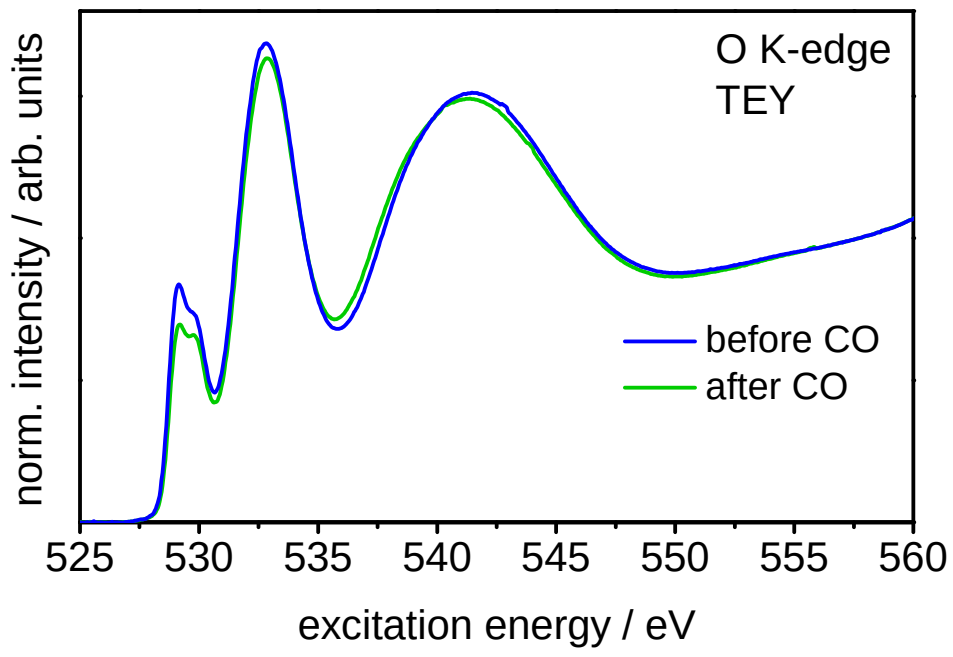

Figure S5: O K-edge of the IrO_x sample measured in total electron yield (TEY) mode before and after CO exposure. Like in AEY mode, the spectra witness a relative decrease of the 529 eV feature compared to the 530 eV feature upon CO dosage.

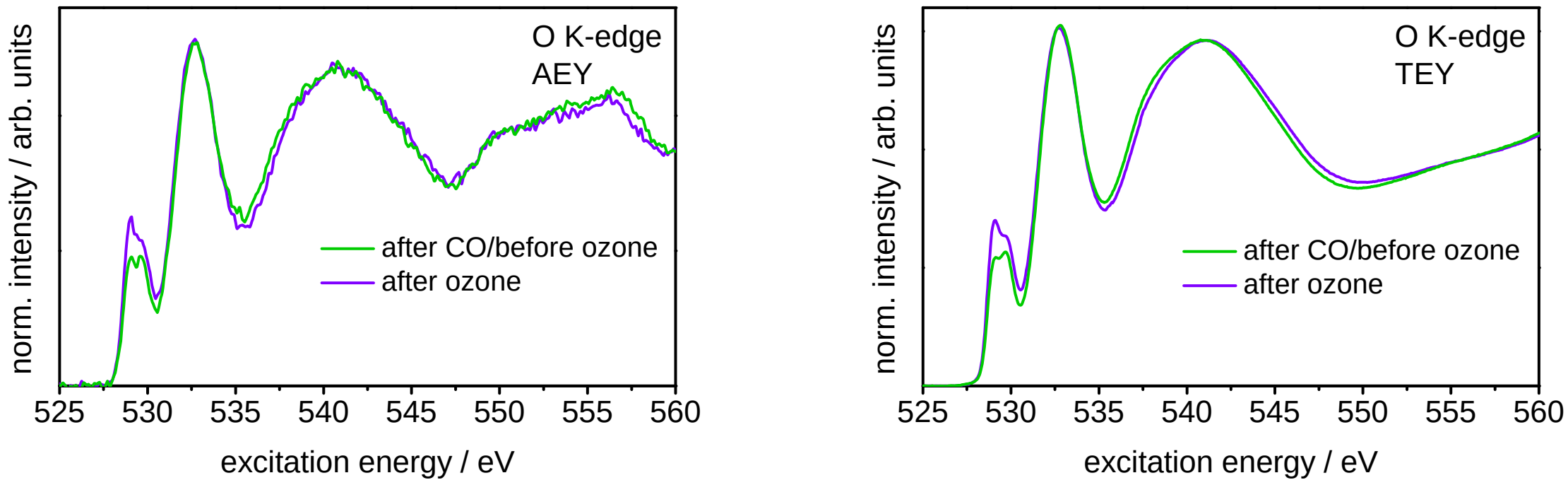

Figure S6: O K-edge of the IrO_x sample measured in Auger electron yield (AEY) (left) and total electron yield (TEY) (right) mode before and after ozone exposure. The spectra witness a relative increase of the 529 eV feature compared to the 530 eV feature upon ozone exposure. The additional contribution in the AEY spectra between 548 eV and 558 eV is due to the S 2p XPS peak interferring with the AEY spectrum.

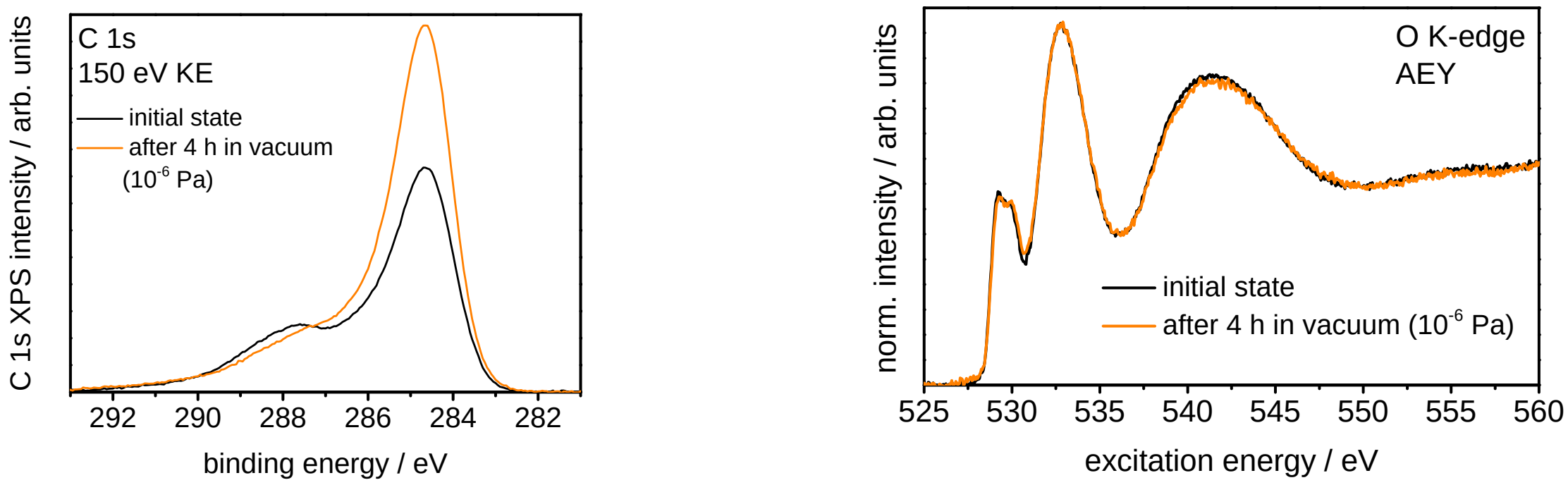

Figure S7: C 1s core level (left) and O K-edge (right) of an IrO_x sample in its initial state and after 4 h in vacuum (base pressure 10^{-6} Pa). The "high" base pressure of the NAP-XPS system leads to graphitic carbon accumulation at the sample surface as evidenced by the increased contribution to the C 1s core level at ≈284.5 eV. The shape of the low excitation energy region of the O K-edge is not affected by sole carbon accumulation.

3 CO titration in SETARAM MS70 Calvet microcalorimeter

The heats of reaction of CO with reactive oxygen species from IrO_x were determined using a SETARAM MS70 Calvet microcalorimeter with a custom-designed high-vacuum and gas-dosing apparatus, described in detail elsewhere.[5] A calibration of the heat-voltage constant of the instrument was done prior to the experiments via the Joule effect. In three runs of the experiment, IrO_x powder volumes between 400 mg and 500 mg were dispensed into the microcalorimeter with a total volume of 274.608 mL. Prior to CO exposure, the powders were dried in vacuum ($<10^{-6}$ Pa) for 72 h to release loosely physisorbed water. When a constant heat signal was reached, the cell was closed. Subsequently, defined amounts of CO 3.7 (Westfalen) were introduced into the initially evacuated measurement volume. The corresponding differential heats of reaction can be calculated by converting the signal area into a heat using the preliminarily determined calibration factor and dividing by the number of involved molecules. For an exothermic process, positive energetic quantities are obtained (calorimetric sign criterion). Figures S8 to S10 show the time-dependent line profiles of the thermosignals for the individual dosing steps performed in the three microcalorimetry experiments. For an easier comparison of the line shapes, the signals are normalized and only shown up to 70 min after the initial CO introduction to the microcalorimeter. In the experiment, prior to the following dosing step, we waited for the signal to completely decay to the thermosignal base line, which could take up to 10 h (not shown). In all three experiments, we observe a broadening of the thermosignal with increasing pressure and amounts of reacted CO, as well as a shift of the maximum of the thermosignal to longer times after the initial CO introduction. The latter trend is only reversed towards the end of the experiment, i.e. the last dosing steps. The broadening and tailing of the signal with higher pressures might be an indication for the deeper layer desorption of O^{I-}, liberated by the reaction heat evolved in the surface reaction.

In a post analysis, the gas phase present in the microcalorimeter after experiment 3 was examined. For this post analysis, the closed microcalorimeter container was connected to a Varian CP-4900 micro gas chromatograph (microGC) with 4 channels (channel 1: mole sieve 5A, channel 2:Al_2O_3, channel 3: CP-Sil 19CB, channel 4: PoraPlot Q). After opening the connecting valve, the gas volume in the microcalorimeter was flushed out by a He stream of initially 1 mL min^{-1} and subsequently 5 mL min^{-1} (Figure S11). In the effluent gas, we found no residual CO but only CO_2 (see Figure S11). This finding suggests that the vast majority of CO introduced to the measurement volume

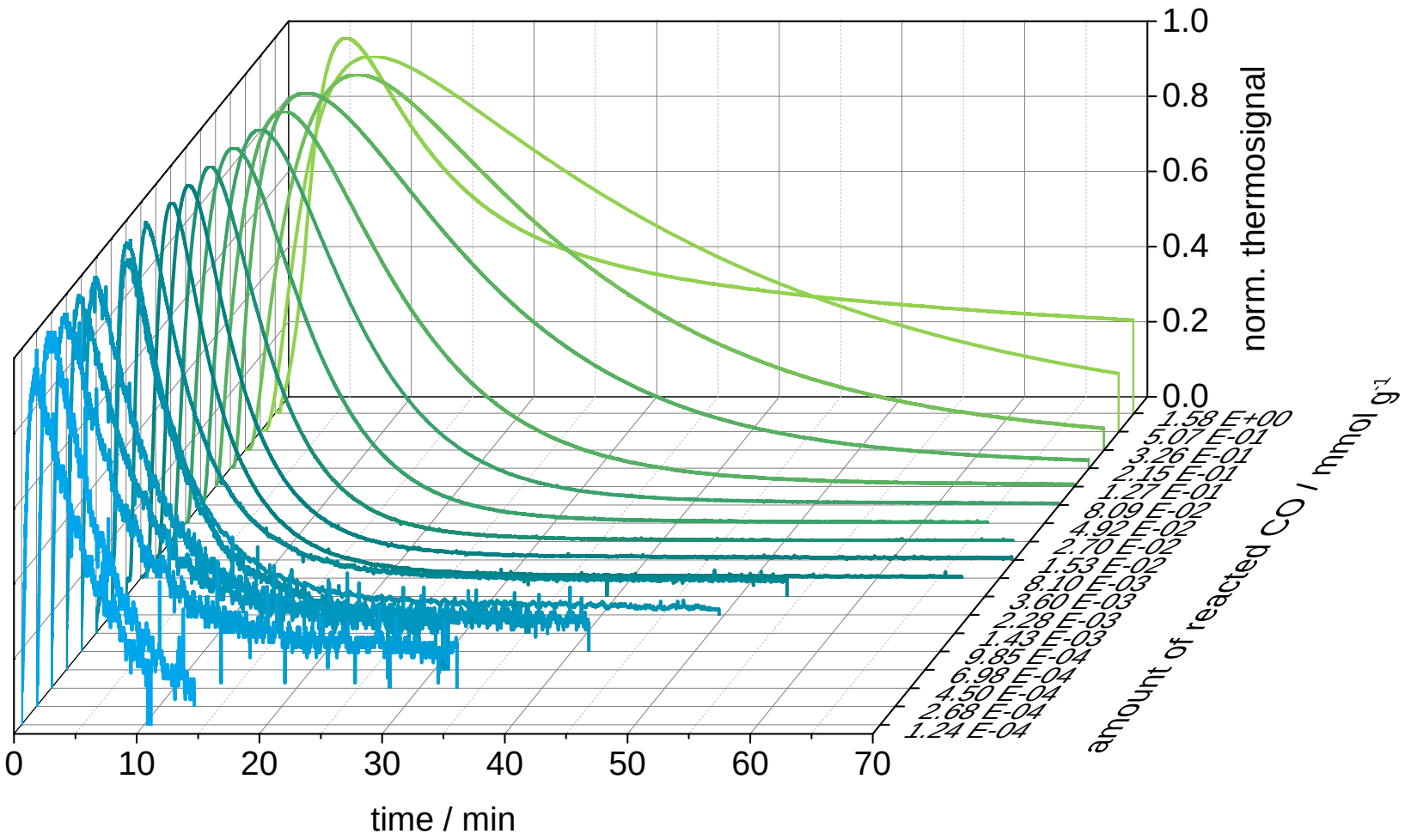

Figure S8: Normalized time-dependent line profiles of the evolved heat in microcalorimetry experiment 1 for different amounts of reacted CO measured at 313 K. At time 0 min the CO was introduced to the microcalorimeter. For better comparison of the line shapes, the recorded heat signals are normalized to 1 and are only shown up to 70 min. In the experiment, the next dosing step was only performed once the thermosignal base line was reached (for higher amounts of reacted CO not shown). The low signal-to-noise ratio for the first dosing steps is due to the very small thermosignals registered.

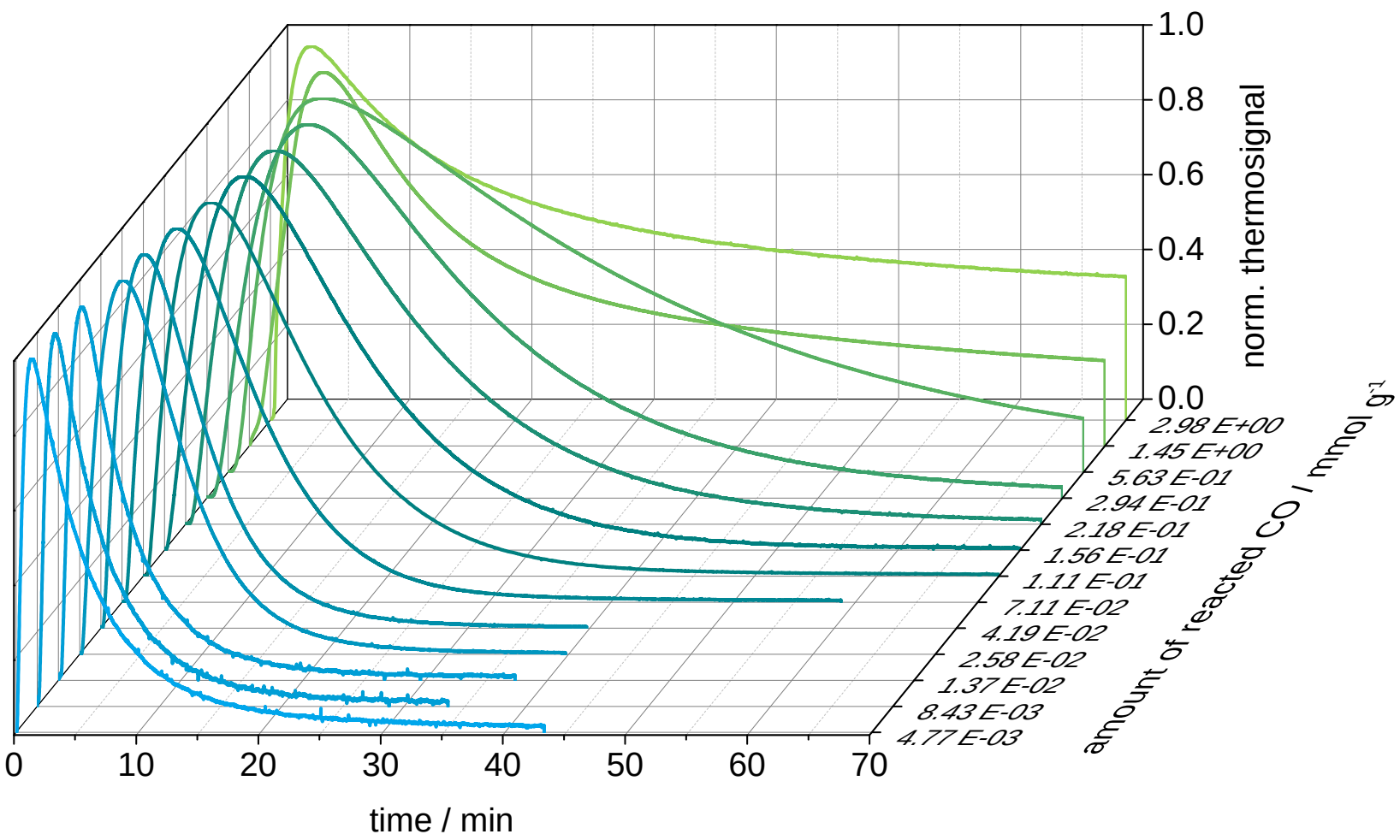

Figure S9: Normalized time-dependent line profiles of the evolved heat in microcalorimetry experiment 2 for different amounts of reacted CO measured at 313 K. At time 0 min the CO was introduced to the microcalorimeter. For better comparison of the line shapes, the recorded heat signals are normalized to 1 and are only shown up to 70 min. In the experiment, the next dosing step was only performed once the thermosignal base line was reached (for higher amounts of reacted CO not shown).

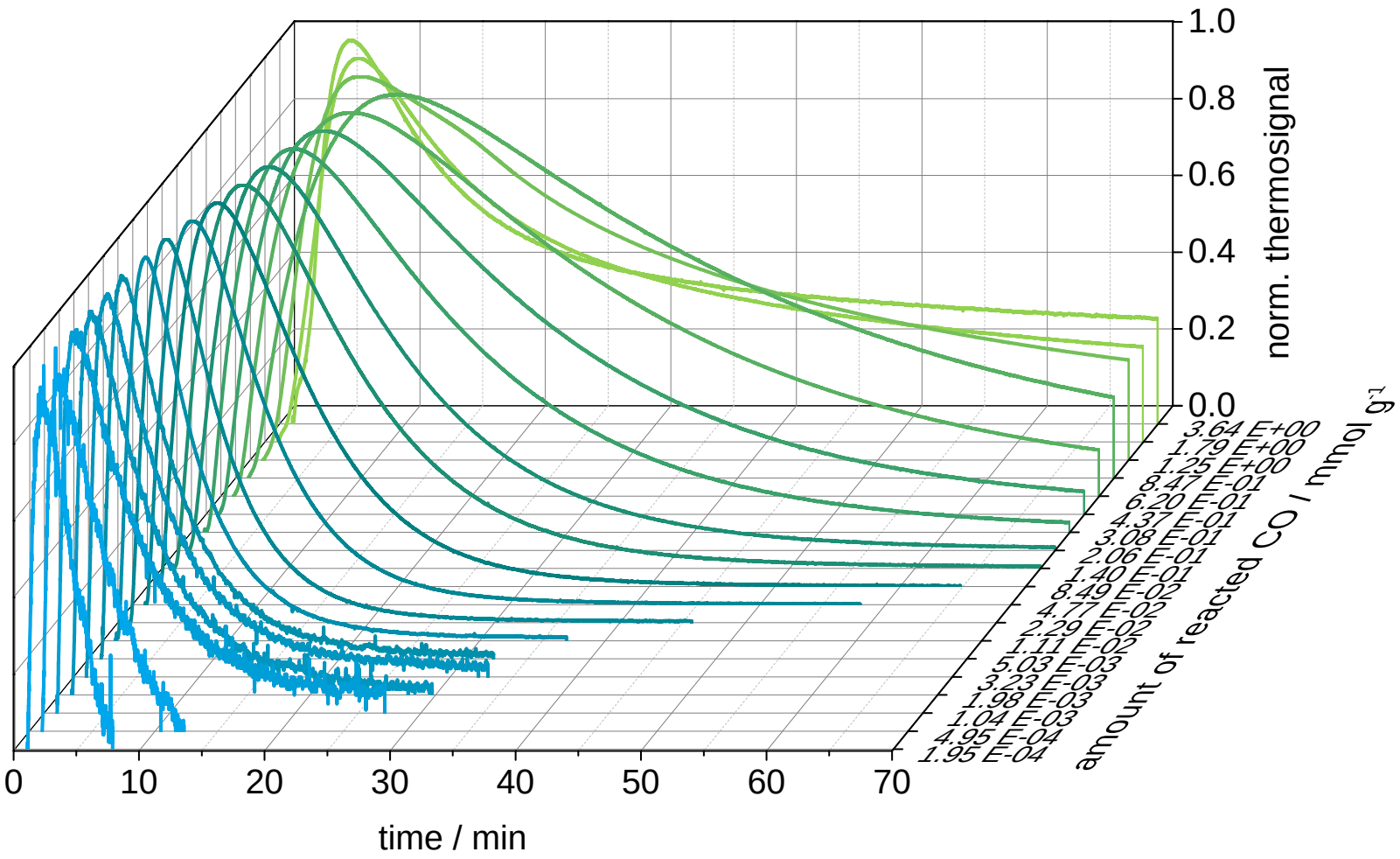

Figure S10: Normalized time-dependent line profiles of the evolved heat in microcalorimetry experiment 3 for different amounts of reacted CO measured at 313 K. At time 0 min the CO was introduced to the microcalorimeter. For better comparison of the line shapes, the recorded heat signals are normalized to 1 and are only shown up to 70 min. In the experiment, the next dosing step was only performed once the thermosignal base line was reached (for higher amounts of reacted CO not shown). The low signal-to-noise ratio for the first dosing steps is due to the very small thermosignals registered.

was consumed by reactive oxygen species of IrO_x. Therefore, in the calculation of the differential heats of reaction, the entire dosed amount of CO was taken into account. There might be, of course, some residual, unreacted CO molecules that were possibly not detected by the microGC. Unfortunately, due to the measurement setup, it was not possible to measure the composition of the gas phase after each CO pulse in order to determine the exact number of molecules that reacted in each step. The reported value for the reaction enthalpy is therefore given with a considerable error bar and has to be viewed as a lower limit for the reaction enthalpy per molecule. The large standard deviation of this measurement shows that the equipment was operated at its limits since it is usually used only for monitoring adsorption and not reaction events.

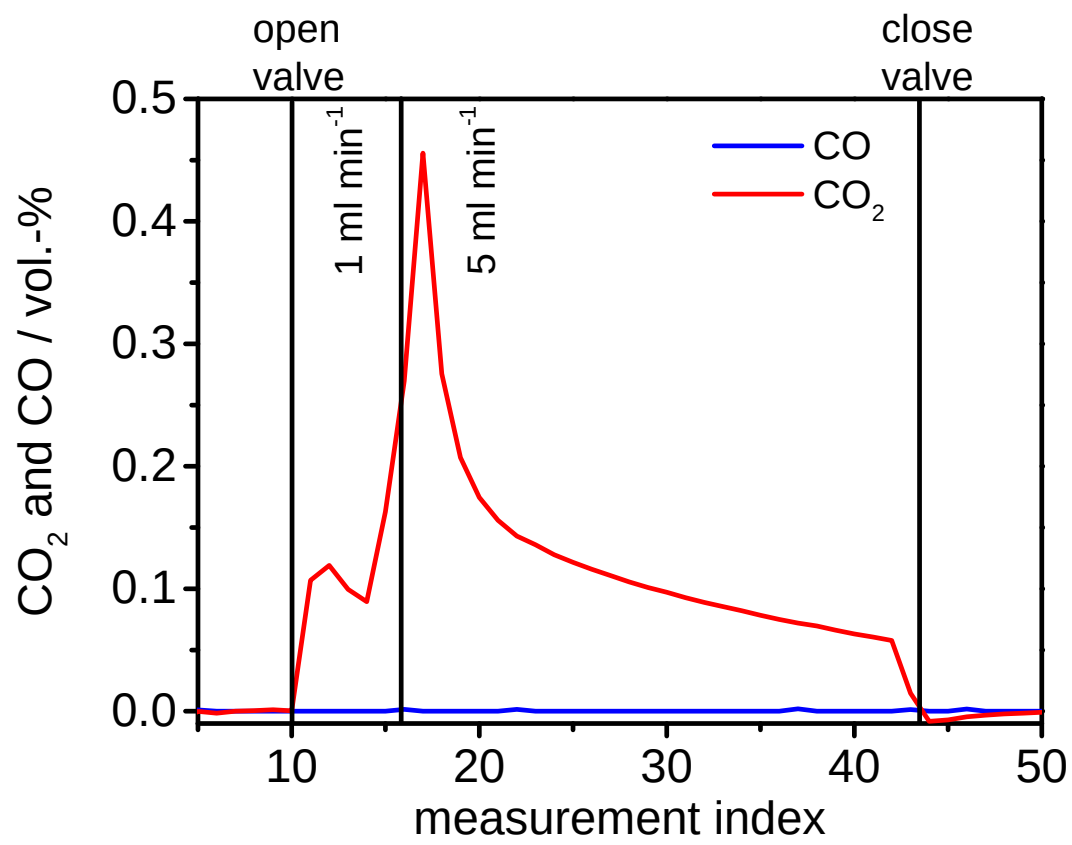

Figure S11: CO_2 and CO concentrations of the post analysis of the gas mixture in the microcalorimeter after CO titration of IrO_x. The profiles confirm a predominant presence of CO_2, which suggests that the dosed CO was completely oxidized.

4 Calculation details

All density functional theory (DFT) calculations were performed using the Quantum ESPRESSO package version 5.3.0.[6] The Perdew, Burke, and Ernzerhof (PBE) exchange and correlation potential[7] was employed as it has been shown to recover the correct ground state electronic structure of IrO_2[8] and has been used to effectively describe the defect states present in the amorphous IrO_x.[1] The present calculations were performed using scalar relativistic corrections and without spin polarization. Ultrasoft pseudopotentials taken from the PS library[9] were employed for all total energy calculations. Two Gauge-Including Augmented Wave (GIPAW) projectors were included for the l=1 channel of the oxygen pseudopotential to allow us to compute the O K-edge spectra. A plane wave basis set was employed with a kinetic energy cutoff of 30 Ry and a charge density cutoff of 300 Ry. Bulk calculations were performed using a **k**-point mesh equivalent to (8x8x8) for the 24 atom cell shown in Figure S12 and previously described.[1] Slab calculations were performed using 10 layers of the crystallographic unit cell for the (110) and (113) surface with approximately 15 Å of vacuum separating periodic images and a **k**-point mesh equivalent to (8x4x1) for the surface unit cells. Climbing image nudged elastic band calculations (see below) used the full (113) slab but were performed with only 5 layers of the crystallographic unit cell for reactions on the (110) surface. Marzari-Vanderbilt cold smearing[10] was used with a smearing parameter of 0.01 Ry.
Climbing image nudged elastic band calculations were performed to identify the transition states for the reaction of CO on the (110) and (113) surfaces. These were performed

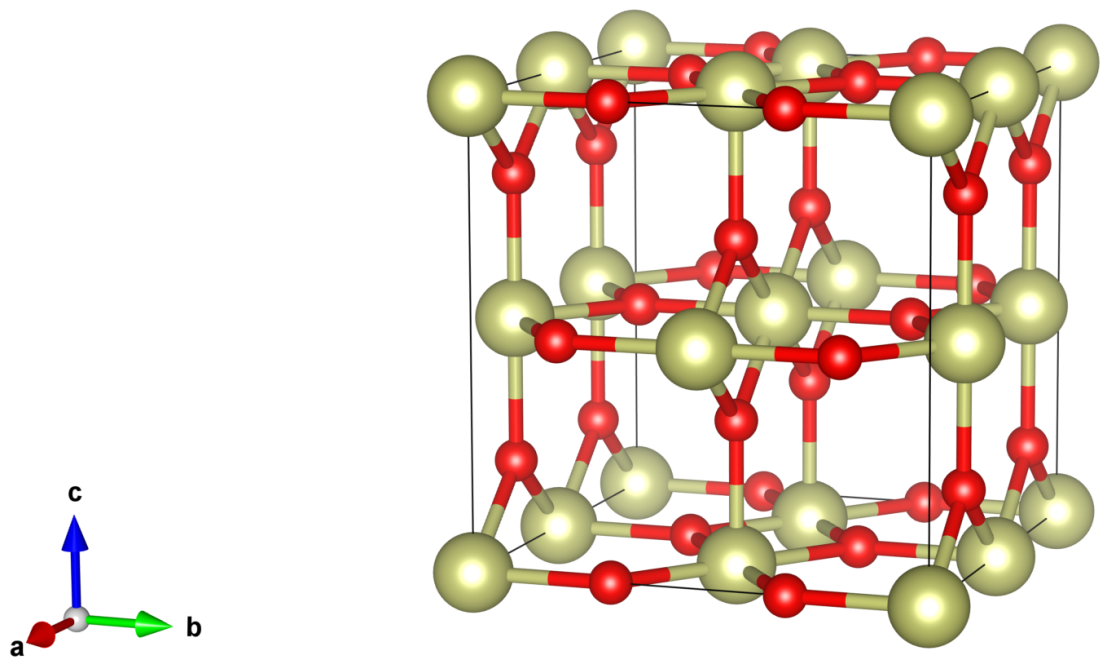

Figure S12: The bulk rutile-type IrO_2 cell used in the present work is identical to that employed in Pfeifer *et al.*[1]

using 8 images of the system until the forces on the images were less than 0.05 eV Å^{-1}.The ΔSCF (self-consistent field) method was used to compute core level binding energies (BE) to ensure both initial and final state effects were captured.[11] The resultant relative O 1s BEs were shifted to absolute BEs using a reference calculation on a (4x4x4) supercell of IrO_2. We took the measured O 1s BE of the reference system as 530.0 eV. Previously,[1] we have verified that the relative BEs in IrO_2 were converged to better than 0.1 eV with our computational setup.

4.1 O K-edge spectra

Oxygen K-edge spectra were computed in two ways, which proved to be nearly identical, see Figure S13. In the first we approximated the X-ray absorption process using a one-electron Fermi's golden rule expression as implemented in the XSpectra package.[12,13] Here we neglected the core-hole and used the ground state results from the USPP calculations. We previously found this approach to give good agreement with experiment for rutile-type IrO_2 when the computed spectra were convoluted using a Lorentzian with an energy dependent linewidth, $\Gamma(E) = \Gamma_0 + \Gamma(E)$, to account for lifetime broadening.[1]
In an effort to ensure this one-electron approximation remains valid for the defect structures giving rise to the O^{I-} state, we also employed a resolvent-based Bethe-Salpeter Equation (BSE) approach to capture the screened core-hole potential and electron-hole dynamics.[14,15] These calculations were carried out by combining the Kohn-Sham wavefunctions from QE (Quantum Espresso) with the NIST core-level BSE solver (NBSE)

through the OCEAN package.[16,17] For these calculations, however, we employed norm conserving pseudopotentials generated with the FHI98PP package.[18] The exchange and correlation was treated with the local density approximation (LDA) based on Perdew and Wang's parameterization of Ceperely and Alder's data.[19] We found the results were converged using a kinetic energy cutoff of 100 Ry (400 Ry) for wavefunctions (charge density) with ground and final state **k**-point mesh equivalent to those used in the total energy calculations. Methfessel-Paxton smearing[20] was used with a width of 0.002 Ry during the self-consistent field calculations. The screening calculations were performed including bands to capture states over 100 eV above the Fermi energy. All spectra were broadened with a Lorentzian with a constant width of 0.2 eV to account for lifetime broadening.

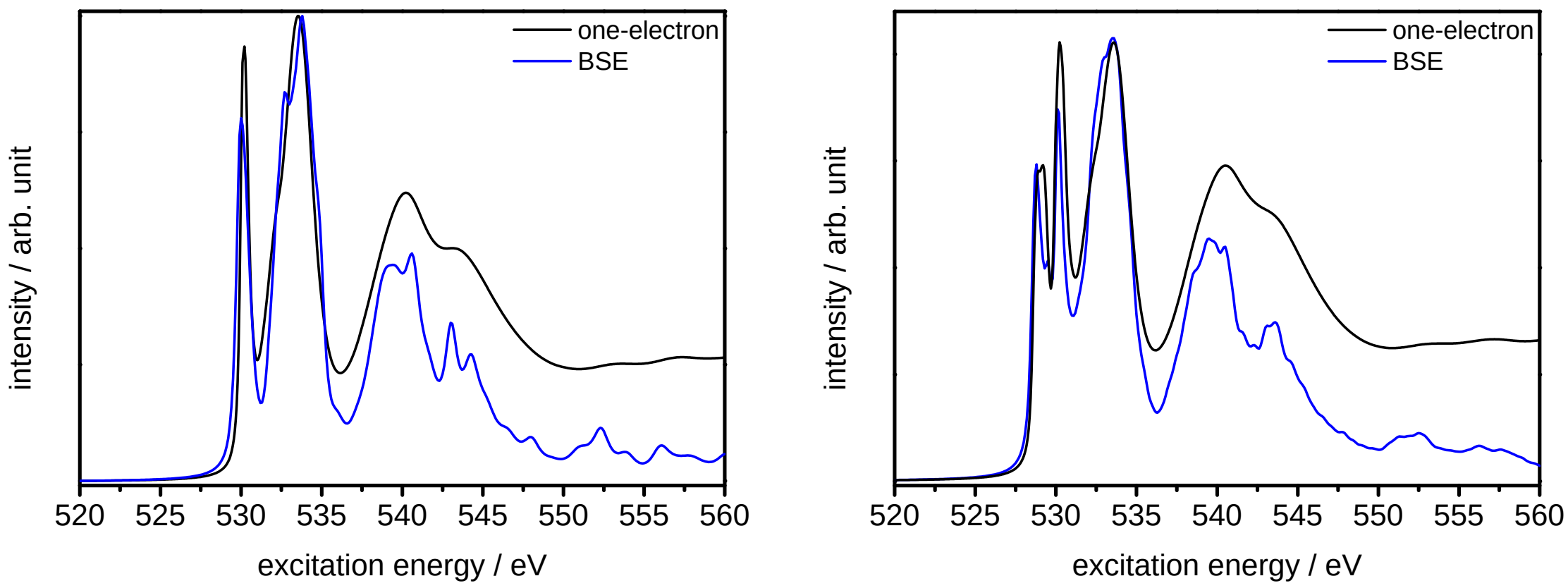

Figure S13: O K-edges computed using the BSE (blue curve) and one-electron (black curve) approaches for rutile-type IrO_2 (left) and O^{I-} and O^{II-} (right).

For both the BSE and one electron calculations the spectra of the symmetry unique oxygen atoms were aligned relative to one another using their corresponding ΔSCF binding energy. Gaussian broadening (FWHM=0.6 eV) was also introduced to account for instrument resolution and phonon broadening.

Figure S13 shows the BSE and one-electron O K-edge spectra of IrO_2 (left) and the IrO_x-type oxide discussed earlier (right).[1] Both calculations are shown without Gaussian broadening to highlight their differences. The spectrum calculated with the one-electron approximation can be seen to capture all the features present in the BSE approach. While there are slight differences, the remarkable agreement is a consequence of the fact that the 1s core hole is energetically isolated from other states on the atom, which tends to diminish the importance of core-hole dynamics. As a result, the primary differences in the spectra are due to the two treatments of lifetime broadening and core-hole potential,

e.g. linear response within the random phase approximation for the BSE calculation compared to the DFT ground state without a core hole.

4.2 Heats of reaction

By assuming that the reactant CO and product CO_2 are gas-phase species, as indicated by gas-phase analysis, the heat of reaction can be computed as:

$$\Delta H_{\mathrm{rxn}} = E_{\mathrm{IrO_x}} - E_{\mathrm{IrO_x-O^*}} + E_{\mathrm{CO_2}} - E_{\mathrm{CO}} \tag{1}$$

where $E_{\mathrm{IrO_x}}$ is the total energy of the IrO_x system missing one oxygen, $E_{\mathrm{IrO_x-O^*}}$ is the total energy of the parent IrO_x-O* system, and $E_{\mathrm{CO_2}}$ (E_{CO}) is the total energy of an isolated CO_2 (CO) molecule computed at the Γ-point in a 30 Å x 30 Å x 30 Å box. The $E_{\mathrm{CO_2}} - E_{\mathrm{CO}}$ term is a constant that sets the energy of the oxygen atom in the product. Thus, equation 1 could be rewritten:

$$\Delta H_{\mathrm{rxn}} = E^{f}_{\mathrm{O,vac}} + E^{\mathrm{CO_2}}_{\mathrm{O}} \tag{2}$$

where $E^{f}_{\mathrm{O,vac}}$ is the oxygen vacancy formation energy and $E^{\mathrm{CO_2}}_{\mathrm{O}}$ is the energy of an oxygen atom in CO_2. (Note that the heat of $CO + \frac{1}{2}O_2 \rightarrow CO_2$ was computed to be -311 kJ mol^{-1} compared to the -283 kJ mol^{-1} found experimentally due to the significant errors in reactions with changing bond orders associated with the PBE calculations.[21]) From equation 2 it is clear that the oxygen vacancy formation energy sets the heat of reaction under the assumption that the reactant CO and product CO_2 are gas-phase species.

We computed the heat of reaction by equation 2 for oxygen from the 24 atom bulk rutile-type IrO_2 cell shown in Figure S12, both with and without an Ir vacancy. In the case of the Ir vacancy we considered the under coordinated and fully coordinated O atoms. We also examined the same vacancy structure with two hydroxyls in place of two under coordinated O atoms. For surfaces we examined bridging oxygen, oxygen on the coordinatively unsaturated sites (CUS), and oxygen in the plane of a (2x1) (110) surface both with and without an Ir vacancy in the surface, see Figure S14. Inspection of the figure reveals that the O K-edge of the CUS oxygen has a resonance below any observed experimentally. Whereas, the unoccupied states of bridging oxygen are like that of bulk O^{I-} and those of the oxygen in the (110) plane are like that of O^{II-}. We also considered the heat of reaction of CO with oxygen on a (113) surface of rutile-type IrO_2. For this purpose we took the heat of reaction from the surface.

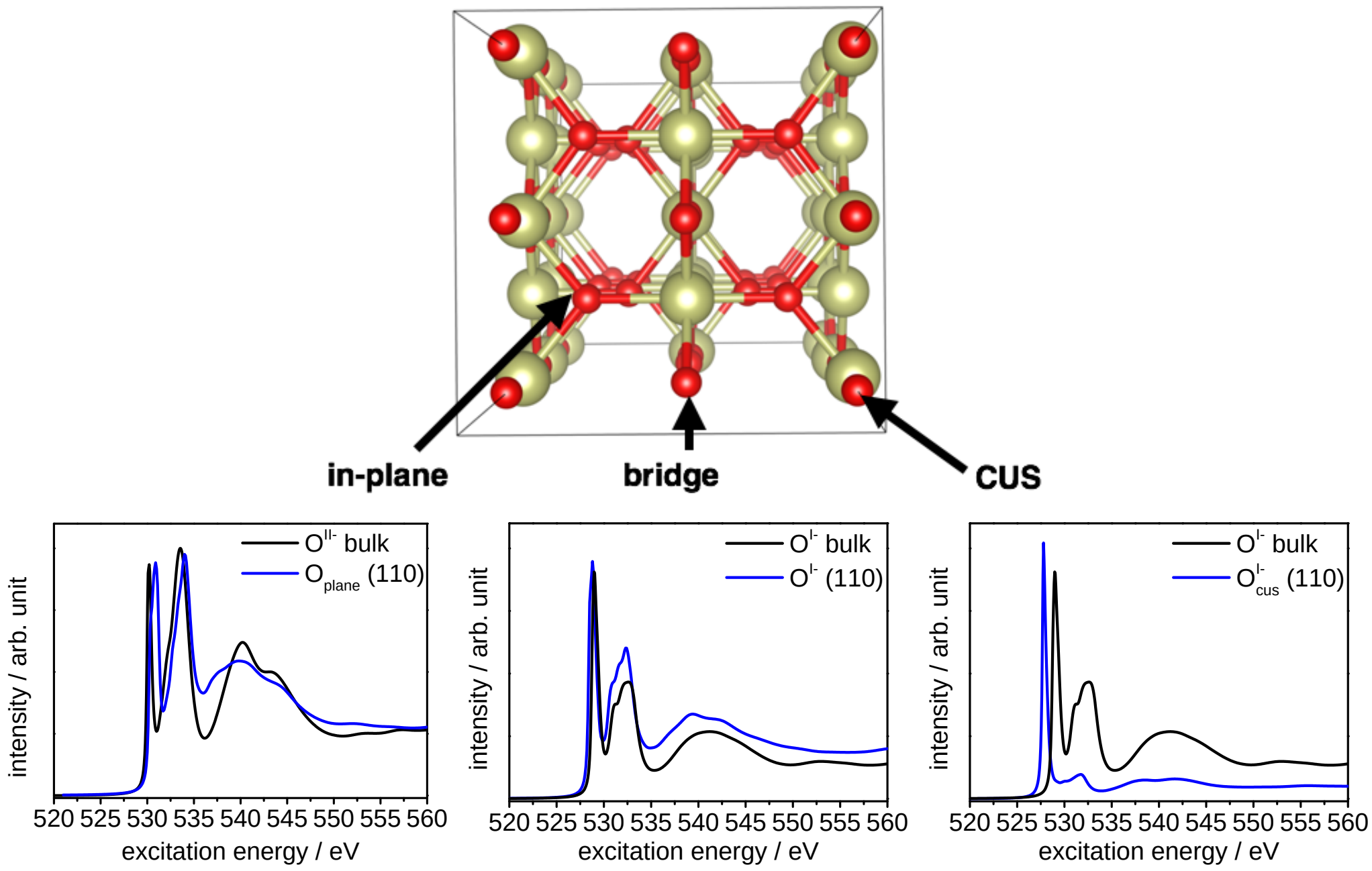

Figure S14: Oxygen sites considered for heat of reaction on pristine (110) surface. The corresponding O K-edge (computed with one-electron approximation) are also shown.

The computed heats of reaction are shown graphically in Figure S15, where they are plotted against the position of the white line of the computed O K-edge spectrum of the corresponding oxygen. A linear trend can be seen for cases where Ir-Ir bond formation does not accompany oxygen removal from the surface, as is the case in the bulk and for the (110) surface. The more open (113) surface allows significant surface rearrangement after oxygen abstraction, which, in some cases, is resulting in the formation of Ir-Ir bonding, see for example Figure S16. The resultant drop in energy leads to points that lie off the linear trend in Figure S15. These points are shown as open circles.

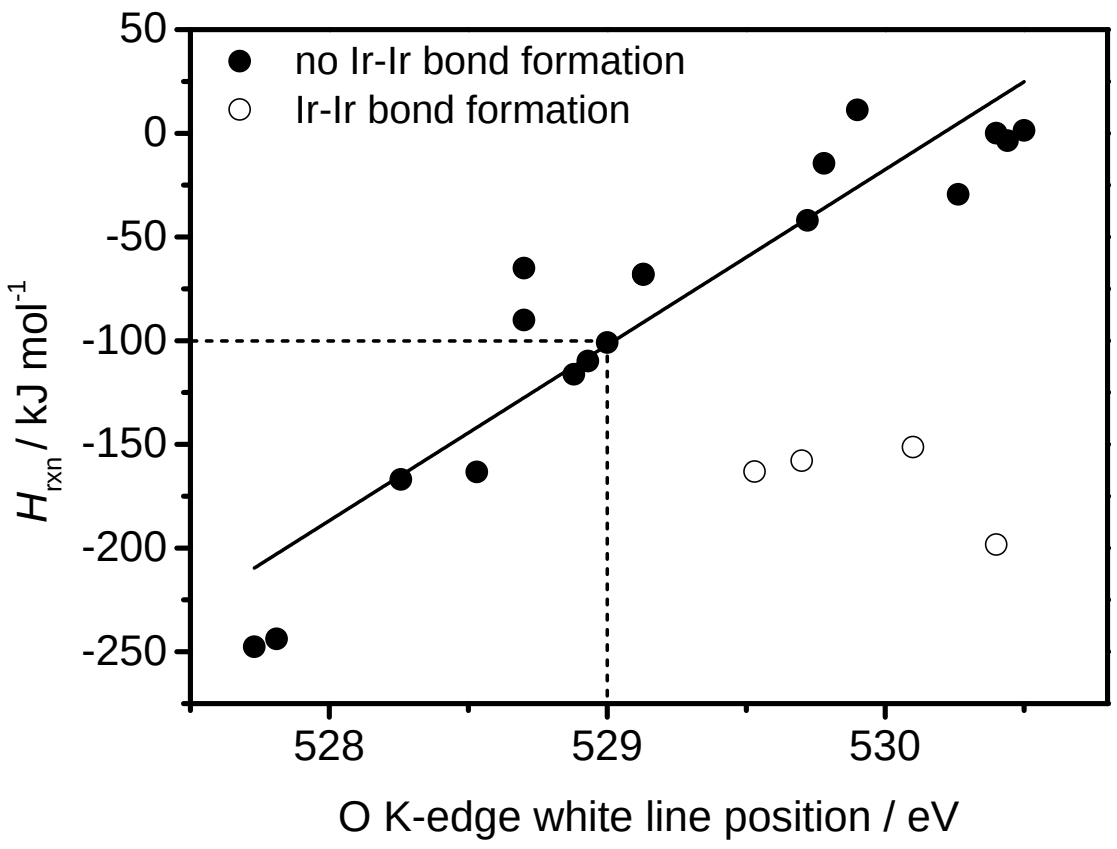

Figure S15: Correlation between the approximate position of the white line in the computed O K-edge spectrum of an oxygen species and its calculated heat of reaction with CO. Solid circles show results when oxygen loss does not lead to Ir-Ir bond formation, empty circles show results when Ir-Ir bonds are formed after oxygen loss. The dashed line shows the 529 eV excitation energy oxygen is predicted to have a heat of reaction of ≈100 kJ mol^{-1} if no additional Ir-Ir bonds are formed after reaction.

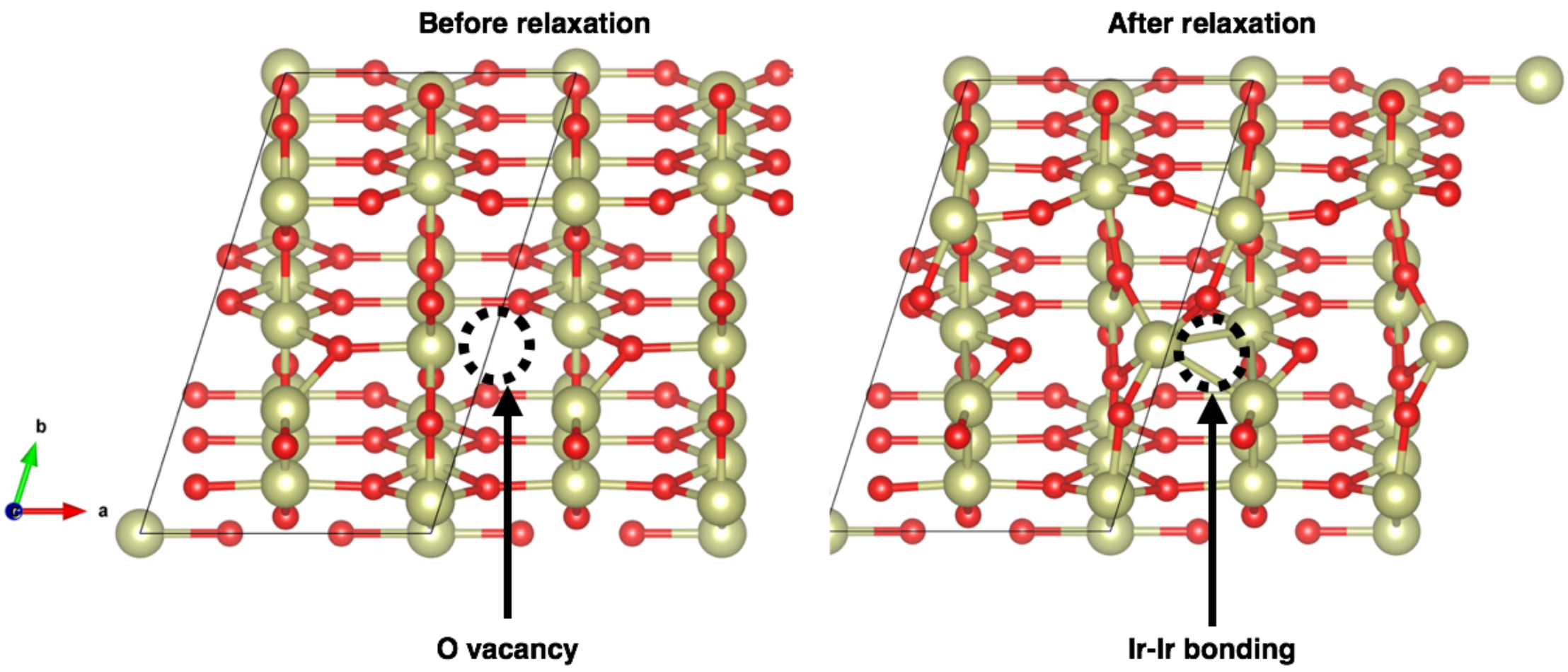

Figure S16: Example of Ir-Ir bond formation during ionic relaxation of (113) surface after O vacancy formation.

4.3 Reactions on (110)

We considered the reaction of $CO_{(g)}$ with O on the (110) CUS, bridge, and lattice sites. In this case, we used the fully oxidized surface, as shown in Figure S14. However, we employed a (1x1) cell for computational efficiency.

Reaction with the CUS oxygen had a barrier of 158 kJ mol^{-1} and a heat of reaction of -245 kJ mol^{-1}. The high barrier may be tied to the symmetry constraint imposed on the

incoming CO, e.g. the CO axis was aligned with the Ir-O_{CUS} axis. Because we saw no spectroscopic evidence for CUS oxygen we did not explore this further.
Conversely, $CO_{(g)}$ reaction with the bridging oxygen had a barrier of 15 kJ mol^{-1} and heat of 87 kJ mol^{-1}. We could not identify a minimum energy path for reaction with the in-plane oxygen.
Thus, only the O^{I-} like species was found to react with gas-phase CO.

4.4 Reactions on (113)

We considered the reaction of CO with oxygen on a partially reduced (113) surface, Figures S17 and S18. The surface has two types of under coordinated Ir atoms that can adsorb gas-phase CO, one is coordinated by 3 oxygen atoms and one by 4 oxygen atoms. Of these, a 4-fold site binds CO the strongest, at 237 kJ mol^{-1}. Thus, we explored the reaction of CO with surface oxygen from this site.

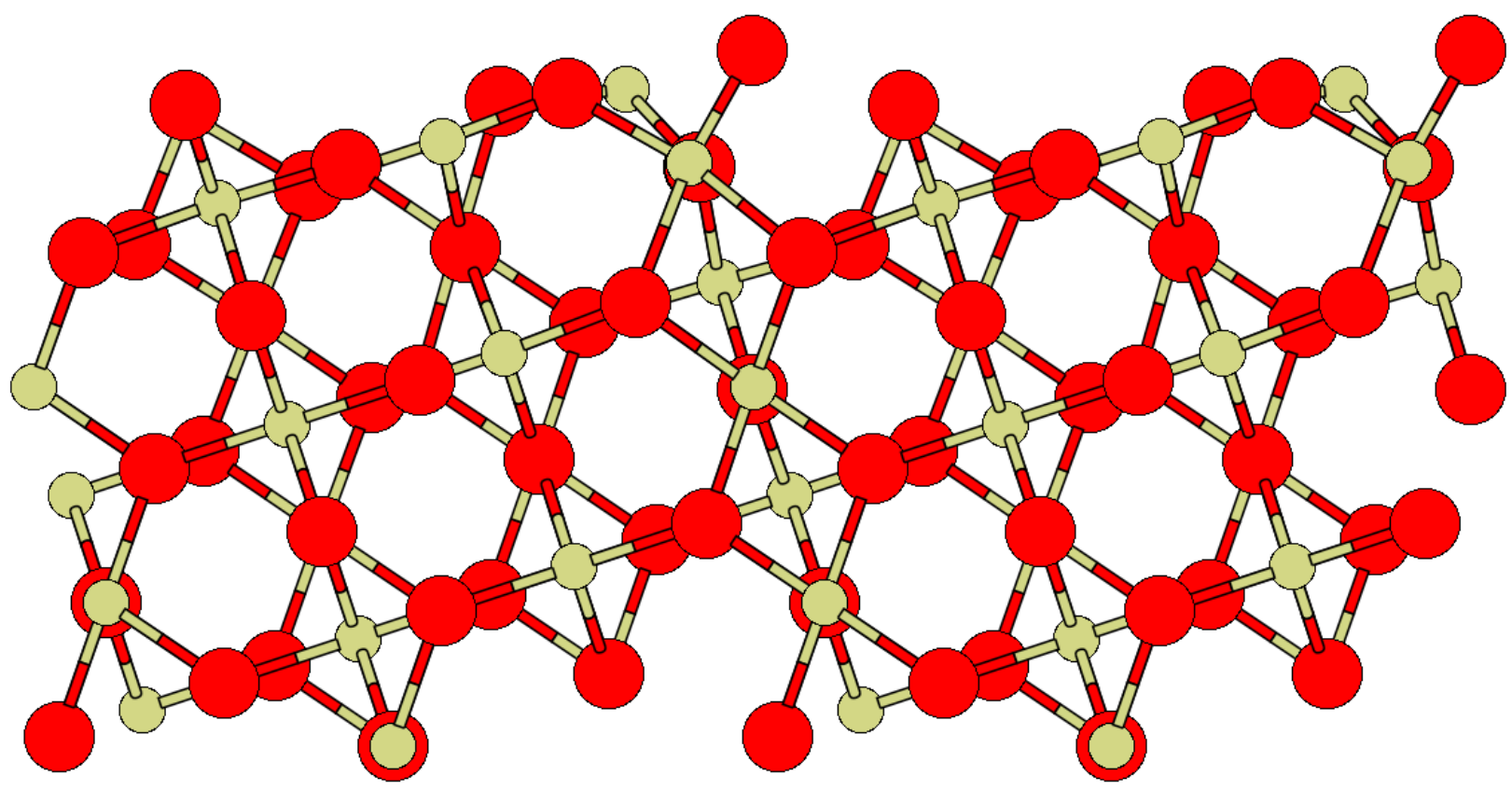

Figure S17: Side view of the (113) surface used in this work. The Ir atoms are shown as yellow spheres and the oxygen as red.

The carbon side of the CO adsorbed on 4-fold site is 3 Å from an O^{I-} site, where the nature of the site was determined by BSE calculation, see Figure S19. We saw that after CO adsorption the (113) surface could distort, thereby forming an Ir-Ir bond, see Figure S20. The barrier for this distortion was only 10 kJ mol^{-1}. It is not clear if the distortion is related to CO adsorption or a property of the clean surface. When CO is present the systems total energy is lowered by 97 kJ mol^{-1} through this Ir-Ir bond formation. From this state the barrier for CO_{ads} reaction was 50 kJ mol^{-1}. The product CO_2 was adsorbed by 163 kJ mol^{-1}.

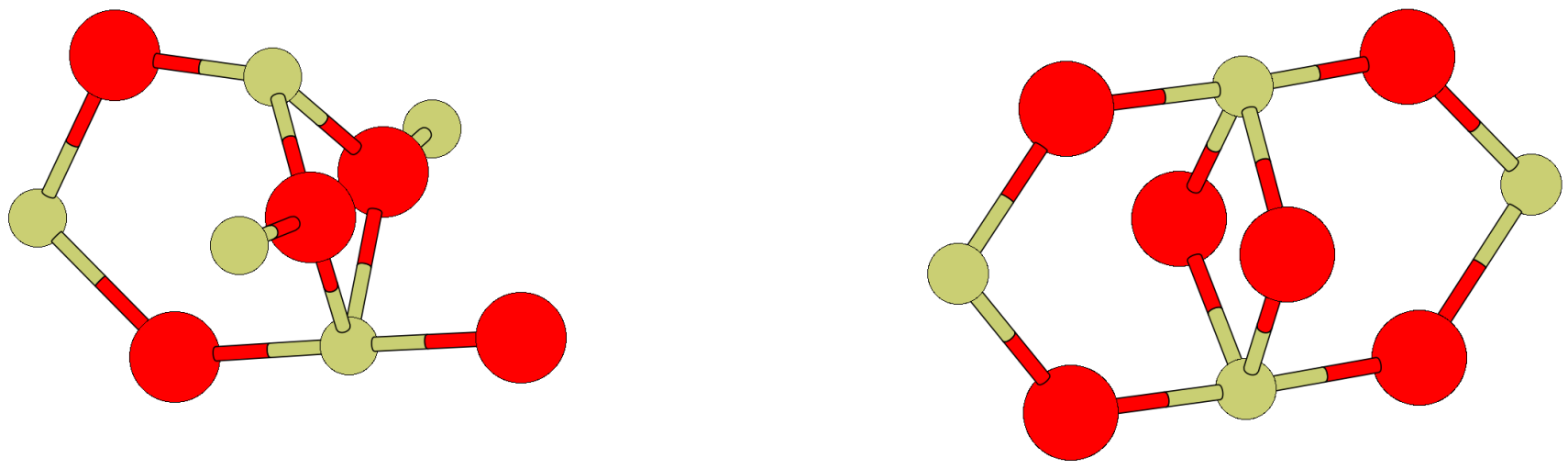

Figure S18: Three (left) and four (right) coordinate Ir atoms on the IrO_2 (113) surface. The Ir atoms are shown as yellow spheres and the oxygen as red.

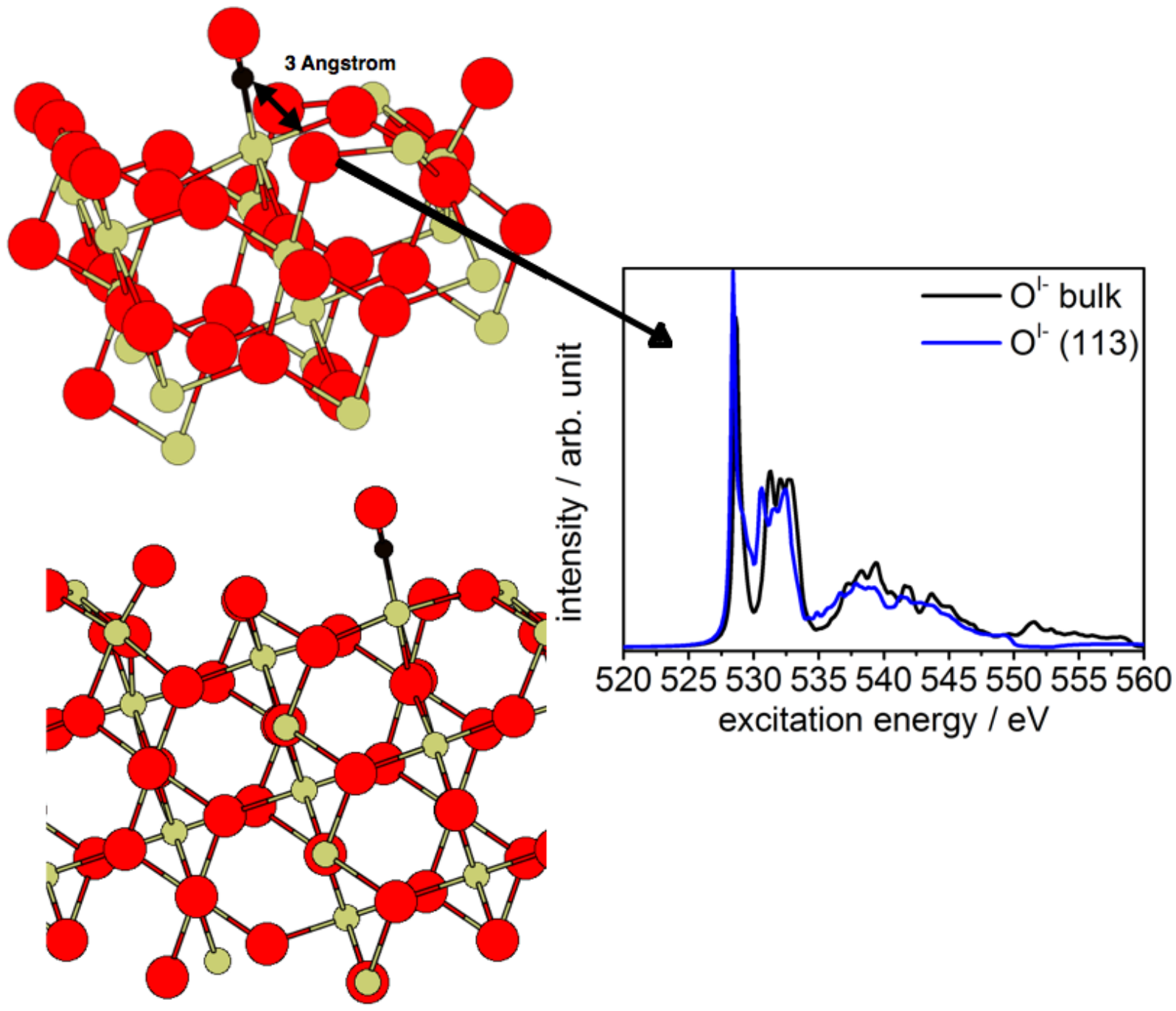

Figure S19: Two views of CO bound to a 4-coordinate Ir site on the (113) surface and the simulated O K-edge of the neighboring O^{I-} species (blue curve) as compared to the bulk O^{I-} species (black curve) without Gaussian broadening.

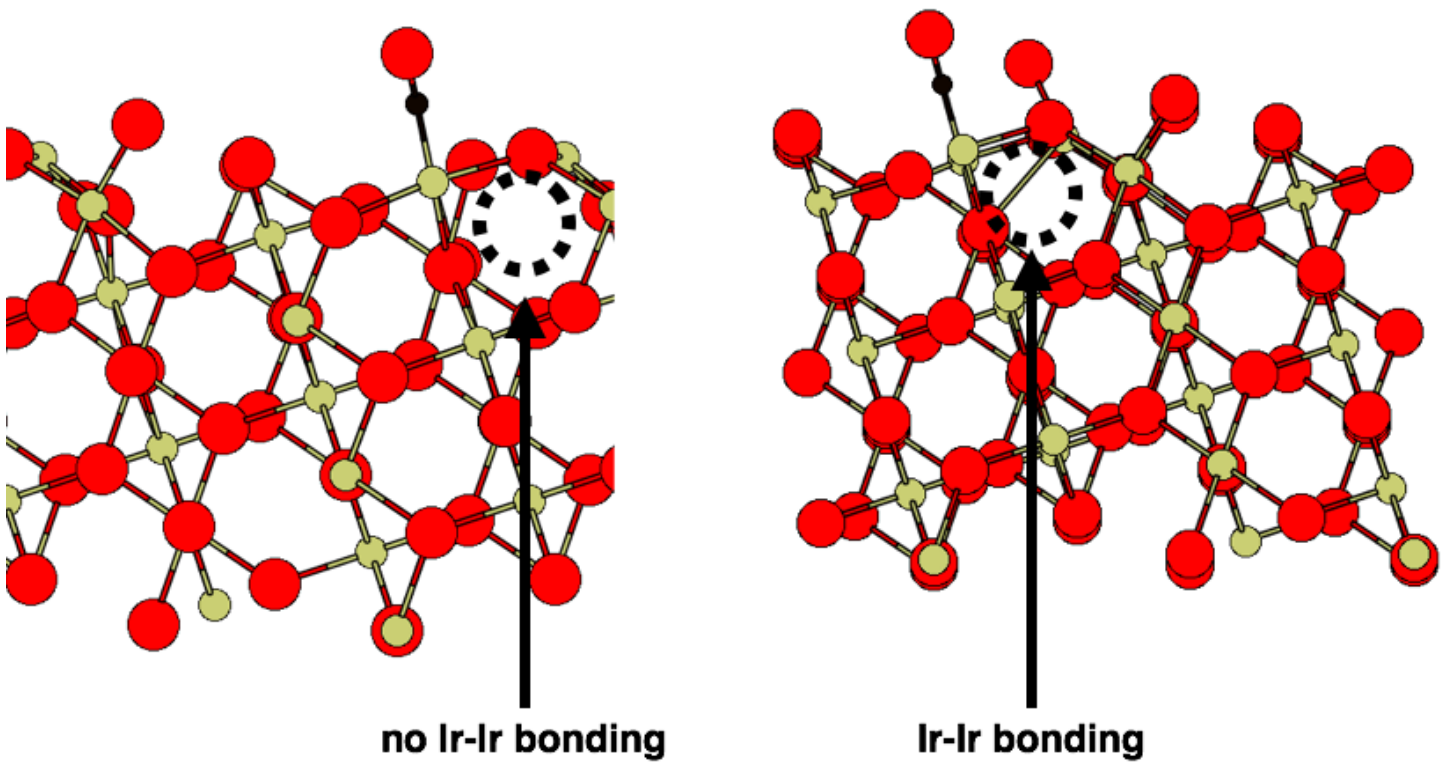

Figure S20: Side view of the (113) surface before and after Ir-Ir bond formation.

References

[1] V. Pfeifer, T. E. Jones, J. J. Velasco Vélez, C. Massué, M. T. Greiner, R. Arrigo, D. Teschner, F. Girgsdies, M. Scherzer, J. Allan, M. Hashagen, G. Weinberg, S. Piccinin, M. Hävecker, A. Knop-Gericke and R. Schlögl, *Phys. Chem. Chem. Phys.*, 2016, **18**, 2292–6.

[2] A. Knop-Gericke, E. Kleimenov, M. Hävecker, R. Blume, D. Teschner, S. Zafeiratos, R. Schlögl, V. I. Bukhtiyarov, V. V. Kaichev, I. P. Prosvirin, A. I. Nizovskii, H. Bluhm, A. Barinov, P. Dudin and M. Kiskinova, in *Chapter 4 X-Ray Photoelectron Spectroscopy for Investigation of Heterogeneous Catalytic Processes*, ed. C. G. Bruce and K. Helmut, Academic Press, 2009, vol. 52, pp. 213–272.

[3] S. Tanuma, C. J. Powell and D. R. Penn, *Surf. Interface Anal.*, 1993, **21**, 165–176.

[4] D. Teschner, A. Pestryakov, E. Kleimenov, M. Hävecker, H. Bluhm, H. Sauer, A. Knop-Gericke and R. Schlögl, *J. Catal.*, 2005, **230**, 186–194.

[5] L. C. Jozefowicz, H. G. Karge and E. N. Coker, *J. Phys. Chem.*, 1994, **98**, 8053–8060.

[6] P. Giannozzi, S. Baroni, N. Bonini, M. Calandra, R. Car, C. Cavazzoni, D. Ceresoli, G. L. Chiarotti, M. Cococcioni, I. Dabo, A. D. Corso, S. d. Gironcoli, S. Fabris, G. Fratesi, R. Gebauer, U. Gerstmann, C. Gougoussis, A. Kokalj, M. Lazzeri, L. Martin-Samos, N. Marzari, F. Mauri, R. Mazzarello, S. Paolini, A. Pasquarello, L. Paulatto, C. Sbraccia, S. Scandolo, G. Sclauzero, A. P. Seitsonen, A. Smogunov, P. Umari and R. M. Wentzcovitch, *J. Phys.: Condens. Matter*, 2009, **21**, 395502.

[7] J. P. Perdew, K. Burke and M. Ernzerhof, *Phys. Rev. Lett.*, 1997, **78**, 1396–1396.

[8] Y. Ping, G. Galli and W. A. Goddard, *J. Phys. Chem. C*, 2015, **119**, 11570–11577.

[9] A. Dal Corso, *Comp. Mater. Sci.*, 2014, **95**, 337–350.

[10] N. Marzari, D. Vanderbilt, A. De Vita and M. C. Payne, *Phys. Rev. Lett.*, 1999, **82**, 3296–3299.

[11] E. Pehlke and M. Scheffler, *Phys. Rev. Lett.*, 1993, **71**, 2338–2341.

[12] M. Taillefumier, D. Cabaret, A. M. Flank and F. Mauri, *Phys. Rev. B*, 2002, **66**, 195107.

[13] C. Gougoussis, M. Calandra, A. P. Seitsonen and F. Mauri, *Phys. Rev. B*, 2009, **80**, 075102.

[14] E. L. Shirley, *J. Electron Spec. Rel. Phenom.*, 2004, **136**, 77–83.

[15] J. A. Soininen and E. L. Shirley, *Phys. Rev. B*, 2001, **64**, 165112.

[16] J. Vinson, J. J. Rehr, J. J. Kas and E. L. Shirley, *Phys. Rev. B*, 2011, **83**, 115106.

[17] J. Vinson and J. J. Rehr, *Phys. Rev. B*, 2012, **86**, 195135.

[18] M. Fuchs and M. Scheffler, *Comp. Phys. Comm.*, 1999, **119**, 67–98.

[19] J. P. Perdew and Y. Wang, *Phys. Rev. B*, 1992, **45**, 13244–13249.

[20] M. Methfessel and A. T. Paxton, *Phys. Rev. B*, 1989, **40**, 3616–3621.

[21] I. Grinberg, Y. Yourdshahyan and A. M. Rappe, *J. Chem. Phys.*, 2002, **117**, 2264–2270.
